# Canagliflozin Alleviates Diabetic Glomerular Endothelial Injury via Melibiose in a Microbiota‐Dependent Manner

**DOI:** 10.1002/advs.202517222

**Published:** 2026-05-27

**Authors:** Wei Zhang, Yi Song, Changkun Li, Yuanyuan Luo, Mingwei Shao, Feng Guo, Fangyi Wei, Xunjie Fan, Wenwen Guo, Fengmei Xu, Yanhong Sang, Dongming Zhang, Yanhong Zhou, Lianwei Wang, Zhiqiang Kang, Yingjun Yang, Chunhua Song, Yanxia Liu, Xiaojun Ma, Jiao Wang, Chong Li, Shengnan Ma, Lin Zhao, Zhi Qin, Guolan Xing, Qiubo Zhao, Jun Li, Shumin Song, Dan Zhao, Ting Huang, Qingzhu Wang, Yanyan Zhao, Guijun Qin

**Affiliations:** ^1^ Department of Endocrinology and Metabolism The First Affiliated Hospital of Zhengzhou University Zhengzhou China; ^2^ Tianjian Laboratory of Advanced Biomedical Sciences Zhengzhou China; ^3^ Department of Endocrine and Metabolic Diseases Shanghai Institute of Endocrine and Metabolic Diseases Ruijin Hospital Shanghai Jiao Tong University School of Medicine Shanghai China; ^4^ Department of Endocrinology and Metabolism Hebi Coal Industry (Group) Co., Ltd General Hospital Hebi China; ^5^ Department of Endocrinology and Metabolism The Fifth Affiliated Hospital of Zhengzhou University Zhengzhou China; ^6^ Department of Endocrinology and Metabolism The Second Affiliated Hospital of Zhengzhou University Zhengzhou China; ^7^ Department of Endocrinology Xinxiang Central Hospital Xinxiang China; ^8^ Department of Endocrinology Zhumadian Central Hospital Zhumadian China; ^9^ Department of Endocrinology and Metabolism Zhengzhou Central Hospital Zhengzhou China; ^10^ Department of Endocrinology and Geriatrics The Seventh People's Hospital of Zhengzhou Zhengzhou China; ^11^ Department of Epidemiology and Statistics College of Public Health Zhengzhou University Zhengzhou China; ^12^ Department of Thoracic Surgery The First Affiliated Hospital of Zhengzhou University Zhengzhou China; ^13^ Department of Nephrology The First Affiliated Hospital of Zhengzhou University Zhengzhou China; ^14^ Department of Nuclear Medicine The First Affiliated Hospital of Zhengzhou University Zhengzhou China; ^15^ Henan Provincial Center For Diabetes Prevention and Control Zhengzhou China

**Keywords:** canagliflozin, diabetic kidney disease, gut‐kidney axis, glomerular endothelial injury, glyoxalase 1, melibiose

## Abstract

Canagliflozin reduces albuminuria in patients with diabetic kidney disease (DKD) beyond its glucose‐lowering effect, but the mechanisms remain unclear. We analyzed 85 patients treated with canagliflozin and 85 controls over 26 weeks to explore whether the gut microbiome and its metabolites contribute to renoprotection. Canagliflozin remodeled the gut microbiota, notably enriching *Roseburia intestinalis* and increasing plasma melibiose levels. In mice, canagliflozin alleviated glomerular endothelial injury and albuminuria. Similar effects were replicated by fecal microbiota transplantation, *Roseburia intestinalis*, or melibiose administration. Mechanistically, melibiose bound to and activated glyoxalase 1, reduced methylglyoxal, and suppressed the AGE‐RAGE pathway, preserving glomerular endothelial integrity. Furthermore, oral melibiose precursor supplementation reduced albuminuria in patients with early‐stage DKD. These findings suggest the involvement of a gut‐kidney axis in the renoprotective effects of canagliflozin and indicate that melibiose may serve as a potential therapeutic strategy for DKD.

## Graphical abstract

Canagliflozin treatment reshapes the gut microbiota in DKD and elevates levels of melibiose, a metabolite derived from *Roseburia intestinalis*. Melibiose directly binds to and enhances the enzymatic activity of glyoxalase 1, leading to decreased methylglyoxal accumulation. This restoration of glyoxalase function helps recover endothelial integrity, reverse glycocalyx degradation, attenuate inflammatory injury, and improve fenestrated endothelial architecture, thereby ameliorating diabetic glomerular endothelial damage.

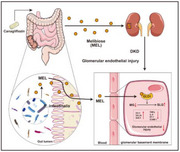

## 1 Introduction

Diabetic kidney disease (DKD) is one of the most common microvascular complications of diabetes and the leading cause of end‐stage renal disease worldwide [1, 2]. A hallmark of DKD is persistent albuminuria [3], which reflects structural and functional damage to the glomerular filtration barrier. In particular, injury to the glomerular endothelium is recognized as an early and pivotal event in DKD pathogenesis [4, 5, 6], contributing to increased permeability and the onset of albuminuria [7]. Despite advances in traditional treatments, a substantial residual risk of DKD progression persists. This underscores the need for novel therapeutic strategies targeting early glomerular endothelial dysfunction directly.

The sodium‐glucose cotransporter 2 inhibitor (SGLT2i) canagliflozin has demonstrated robust renoprotective effects in patients with DKD, significantly reducing albuminuria and attenuating functional decline [8]. Interestingly, beyond its classical action of inhibiting renal glucose reabsorption, canagliflozin has also been shown to modulate intestinal glucose metabolism [9] and reshape gut microbial composition [10], thereby influencing host carbohydrate metabolism [11]. This interplay suggests a potential role of the gut‐kidney axis in mediating canagliflozin therapeutic benefits. The gut microbiota has emerged as a key contributor to DKD pathophysiology [12, 13], producing a spectrum of metabolites that enter systemic circulation and directly influence renal cellular function [14]. As the glomerular endothelium is in continuous contact with the bloodstream, it represents a critical interface for sensing and responding to gut‐derived metabolites [15].

Among the diverse metabolites derived from the gut microbiota, carbohydrate‐based compounds constitute a major class of bioactive molecules that play integral roles in host‐microbiota crosstalk [16]. Melibiose, a disaccharide of microbial origin, has recently gained attention due to its potential beneficial effects, including anti‐inflammatory and antioxidant properties, as well as its prebiotic functions [17, 18, 19, 20]. Nevertheless, the role of melibiose and other microbiota‐derived carbohydrates in glomerular endothelial function and DKD remains largely unexplored.

This study aimed to investigate the regulatory effects of canagliflozin on the gut microbiome and metabolome in both patients and mouse models of DKD, to evaluate the protective role of microbiota‐derived metabolites on glomerular endothelial injury, and to identify the underlying molecular targets. By uncovering the gut‐kidney axis‐mediated crosstalk involved in canagliflozin's antiproteinuric effects, this work offers a new insight for targeted DKD therapies.

## 2 Results

### 2.1 Canagliflozin Reshapes the Gut Microbiota in DKD Patients

**FIGURE 1:**
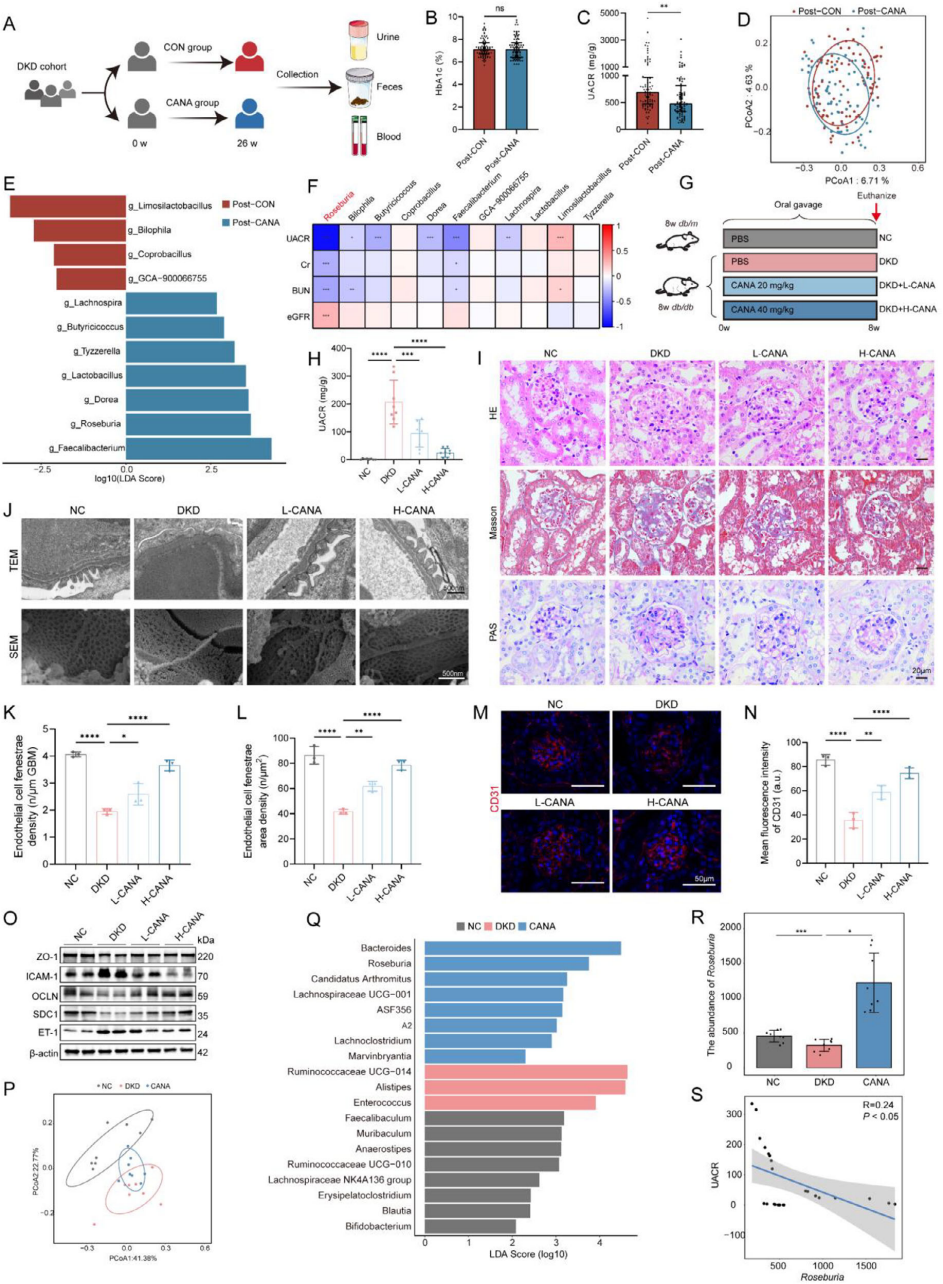
Canagliflozin reshapes the gut microbiota in DKD and ameliorates glomerular endothelial injury. (A) Clinical study flow chart of DKD patients receiving canagliflozin (CANA group, *n* = 85) or control (CON group, *n* = 85). Blood, urine, and fecal samples were collected at baseline and post‐intervention. (B,C) HbA1c (B) and UACR (C) levels in canagliflozin‐treated patients (*n* = 85) and control patients (*n* = 85). (D) Principal coordinate analysis (PCoA) based on Bray‐Curtis distances indicated gut microbiota compositions between the CANA and CON groups. Each dot represents a single sample, red indicates the CON group, and blue indicates the CANA group. (E) Linear discriminant analysis effect size was used to identify differentially abundant taxa between the CON and CANA groups (LDA > 2 and *p* < 0.05). Red and blue indicate genera enriched in the CON and CANA groups, respectively. (F) Pearson's correlations assessing the relationships between the relative abundance of selected bacteria and clinical parameters. (G) Experimental scheme for canagliflozin intervention. Male DKD mice received canagliflozin at 20 or 40 mg kg^−1^, or PBS for 8 weeks, and age‐matched non‐DKD control mice were administered PBS. H) UACR levels in mice (*n* = 8). (I) Representative images of H&E, Masson's trichrome, and PAS staining showing DKD‐associated glomerular pathology. Scale bar, 20 µm. (J) Representative TEM and SEM images showing DKD‐associated glomerular endothelial injury. Scale bar, 500 nm. (K) Morphometric quantification of the endothelial cell fenestration density per unit length, performed on TEM images (*n* = 3). (L) Morphometric quantification of the endothelial cell fenestration area density, performed on SEM images (*n* = 3). M) Representative immunofluorescence images of glomeruli showing CD31 (red) staining for endothelial cells and DAPI (blue) staining for nuclei in DKD mice. Scale bar, 50 µm. (N) Quantitative analysis of CD31 fluorescence intensity in glomeruli of DKD mice (*n* = 3). (O) Representative immunoblotting of ZO‐1, ICAM‐1, OCLN, SDC1, and ET‐1 proteins in the kidney (*n* = 4). (P) PCoA based on Bray‐Curtis distances indicated gut microbiota compositions among the NC, DKD, and CANA groups. Each dot represents an individual mouse (*n* = 8); gray, red, and blue indicate the NC, DKD, and CANA groups, respectively. (Q) Linear discriminant analysis effect size was used to identify differentially abundant taxa among the NC, DKD, and CANA groups (LDA > 2 and *p* < 0.05). (R) Relative abundance of *Roseburia* based on 16S rRNA sequencing in mice (*n* = 8). (S) Linear regression analysis between the relative abundance of *Roseburia* and UACR in NC, DKD, and CANA groups (*n* = 8). Data are presented as median with interquartile range (B, C) and mean ± SD (H, K, L, N, R). Statistical analysis was performed using the Mann–Whitney U tests (B, C) and one‐way ANOVA (H, K, L, N, R). *p < 0.05, **p < 0.01, ***p < 0.001, ****p < 0.0001, ns *p* > 0.05.

We established a multicenter cohort comprising patients receiving canagliflozin treatment and a control group without SGLT2i treatment at baseline (Figure 1A; Figure S1). Despite similar baseline glycated hemoglobin A1c (HbA1c), canagliflozin treatment for 26 weeks significantly reduced urinary albumin‐to‐creatinine ratio (UACR) (Figure 1B,C; Table S1). Detailed information on antihyperglycemic therapy was provided in Table S2, with no significant differences identified between groups at either baseline or the end of follow‐up. 16S rRNA sequencing showed no difference in Chao1 index (richness), but a higher Simpson index (evenness) and distinct microbial profiles (PERMANOVA, *p* = 0.029) in the canagliflozin group (Figure 1D; Figure S2A,B). The *Firmicutes*/*Bacteroidota* (F/B) ratio increased in the canagliflozin group (Figure S2C). At the genus level, microbial composition showed moderate shifts (Figure S2D). Linear discriminant analysis effect size identified several genera that differed significantly between groups (Figure 1E). Notably, the abundance of *Roseburia* correlated with the renal profile, particularly displaying a strong negative correlation with the UACR (Figure 1F).

### 2.2 Canagliflozin Reshapes Gut Microbiota and Alleviates Glomerular Endothelial Injury in DKD Mice

DKD model mice were treated with canagliflozin by daily oral gavage for 8 weeks (Figure 1G). Canagliflozin significantly reduced fasting blood glucose levels, increased body weight (Figure S2E,F), improved UACR (Figure 1H), creatinine and blood urea nitrogen (BUN) (Figure S2G,H), and attenuated glomerular hypertrophy, mesangial matrix deposition, and basement membrane thickening (Figure 1I). Notably, transmission electron microscopy (TEM) and scanning electron microscopy (SEM) revealed restored endothelial fenestration (Figure 1J–L). The expression of the endothelial marker CD31, reduced in DKD mice, was restored by canagliflozin (Figure 1M,N). Additionally, canagliflozin significantly increased the expression of key markers related to endothelial integrity, including zonula occludens‐1 (ZO‐1) and occludin (OCLN), as well as the glycocalyx core protein syndecan‐1 (SDC1). It also reduced the levels of injury markers endothelin‐1 (ET‐1) and intercellular adhesion molecule‐1 (ICAM‐1) (Figure 1O; Figure S2I–M). Gut microbiota profiling demonstrated that canagliflozin treatment increased *Roseburia* and other beneficial taxa, with microbial composition resembling that of healthy controls (Figure 1P–R; Figure S2N,O). Additionally, *Roseburia* abundance remained negatively correlated with UACR in mice (Figure 1S). These results indicate that the gut microbiota, particularly *Roseburia*, may mediate the protective effects of canagliflozin on glomerular endothelial injury.

### 2.3 The Reshaping of the Gut Microbiota by Canagliflozin Ameliorates Diabetic Glomerular Endothelial Injury

**FIGURE 2:**
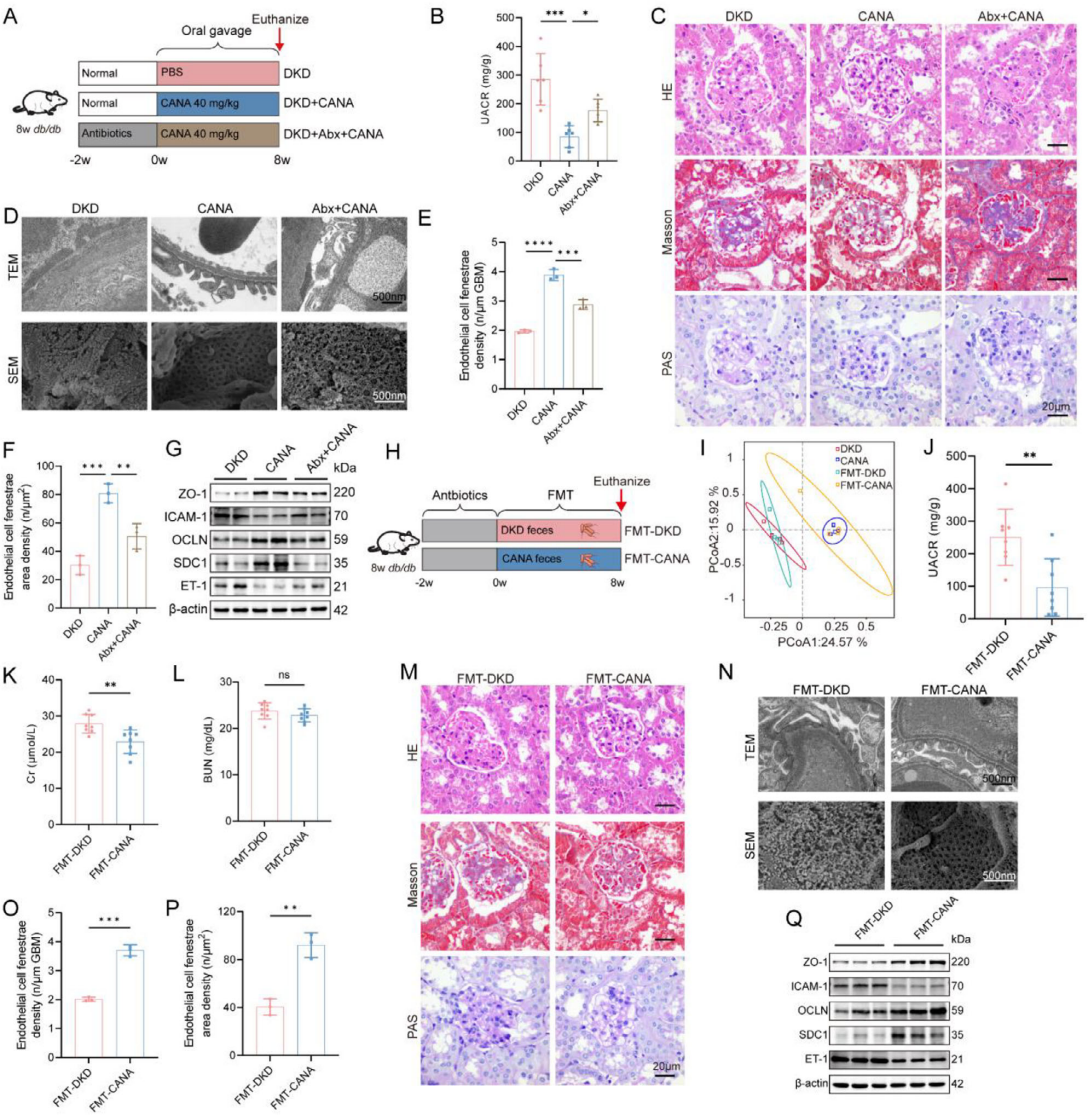
Gut microbiota contributes to the protective effects of canagliflozin on glomerular endothelial injury in DKD mice. (A) Experimental scheme showing the administration of canagliflozin (40 mg kg^−1^) in DKD mice (CANA) and antibiotic cocktail‐induced pseudo‐germ‐free DKD mice (Abx+CANA) for 8 weeks. DKD control mice receiving PBS (DKD). (B) UACR levels in mice (*n* = 6). (C) Representative images of H&E, Masson's trichrome, and PAS staining showing DKD‐associated glomerular pathology. Scale bar, 20 µm. (D) Representative TEM and SEM images showing glomerular endothelial fenestration structures. Scale bar, 500 nm. (E) Morphometric quantification of the endothelial cell fenestration density per unit length, performed on TEM images (*n* = 3). (F) Morphometric quantification of the endothelial cell fenestration area density, performed on SEM images (*n* = 3). (G) Representative immunoblotting of ZO‐1, ICAM‐1, OCLN, SDC1, and ET‐1 proteins in the kidney (*n* = 4). (H) Experimental scheme of fecal microbiota transplantation (FMT). Fecal supernatants from DKD and CANA donor mice were transplanted into antibiotic cocktail‐induced pseudo‐germ‐free DKD recipient mice for 8 weeks. (I) PCoA based on Bray‐Curtis distances showing gut microbiota compositions among donor (DKD and CANA groups) and recipient (FMT‐DKD and FMT‐CANA groups) mice. Each dot represents an individual mouse (*n* = 4); red and blue indicate DKD and CANA donors, green and yellow indicate FMT‐DKD and FMT‐CANA recipients. (J–L) UACR (J), Cr (K), and BUN (L) levels in mice (*n* = 8). (M) Representative images of H&E, Masson's trichrome, and PAS staining showing DKD‐associated glomerular pathology. Scale bar, 20 µm. (N) Representative TEM and SEM images showing glomerular endothelial fenestration structures. Scale bar, 500 nm. (O) Morphometric quantification of the endothelial cell fenestration density per unit length, performed on TEM images (*n* = 3). (P) Morphometric quantification of the endothelial cell fenestration area density, performed on SEM images (*n* = 3). (Q) Representative immunoblotting of ZO‐1, ICAM‐1, OCLN, SDC1, and ET‐1 proteins in the kidney (*n* = 3). Data are presented as mean ± SD (B, E, F, J–L, O, P). Statistical analysis was performed using the one‐way ANOVA (B, E, F) and the unpaired two‐tailed Student's test (J–L, O, P). *p < 0.05, **p < 0.01, ***p < 0.001, ****p < 0.0001, ns *p* > 0.05.

Next, canagliflozin was administered to mice treated with a broad‐spectrum antibiotic cocktail (Abx) (Figure 2A). Two weeks of pretreatment with Abx effectively cleared the gut flora of the mice (Figure S3A). The gut microbiota depletion had no significant effect on blood glucose levels and partially attenuated the modest weight gain induced by canagliflozin (Figure S3B,C). Notably, it markedly weakened the UACR‐lowering effect of canagliflozin (Figure 2B). In addition, the depletion of the gut microbiota also decreased the positive effect of canagliflozin on glomerular endothelial injury in DKD model mice (Figure 2C–G; Figure S3D–H).

Fecal microbiota transplantation (FMT) was performed to further validate the role of canagliflozin‐reshaped microbiota in ameliorating glomerular endothelial injury in DKD mice. Fresh feces from DKD mice receiving canagliflozin or no treatment (donor) were transplanted into the Abx‐treated DKD mice (recipient) (Figure 2H; Figure S3I). The 16S rRNA gene sequencing results showed that the gut microbiota almost completely overlapped in the recipient and donor mice (Figure 2I). Transplantation of the gut microbiota from canagliflozin‐treated mice significantly reduced fasting blood glucose levels (Figure S3J) without affecting body weight (Figure S3K). It also lowered the UACR (Figure 2J) and creatinine (Figure 2K) levels, with no significant change in the BUN level (Figure 2L). Notably, recipient mice exhibited a marked improvement in glomerular endothelial injury following microbiota transfer from canagliflozin‐treated donors (Figure 2M–Q; Figure S3L–P).

### 2.4 Live *R. intestinalis* Alleviates Glomerular Endothelial Injury in DKD Mice

**FIGURE 3:**
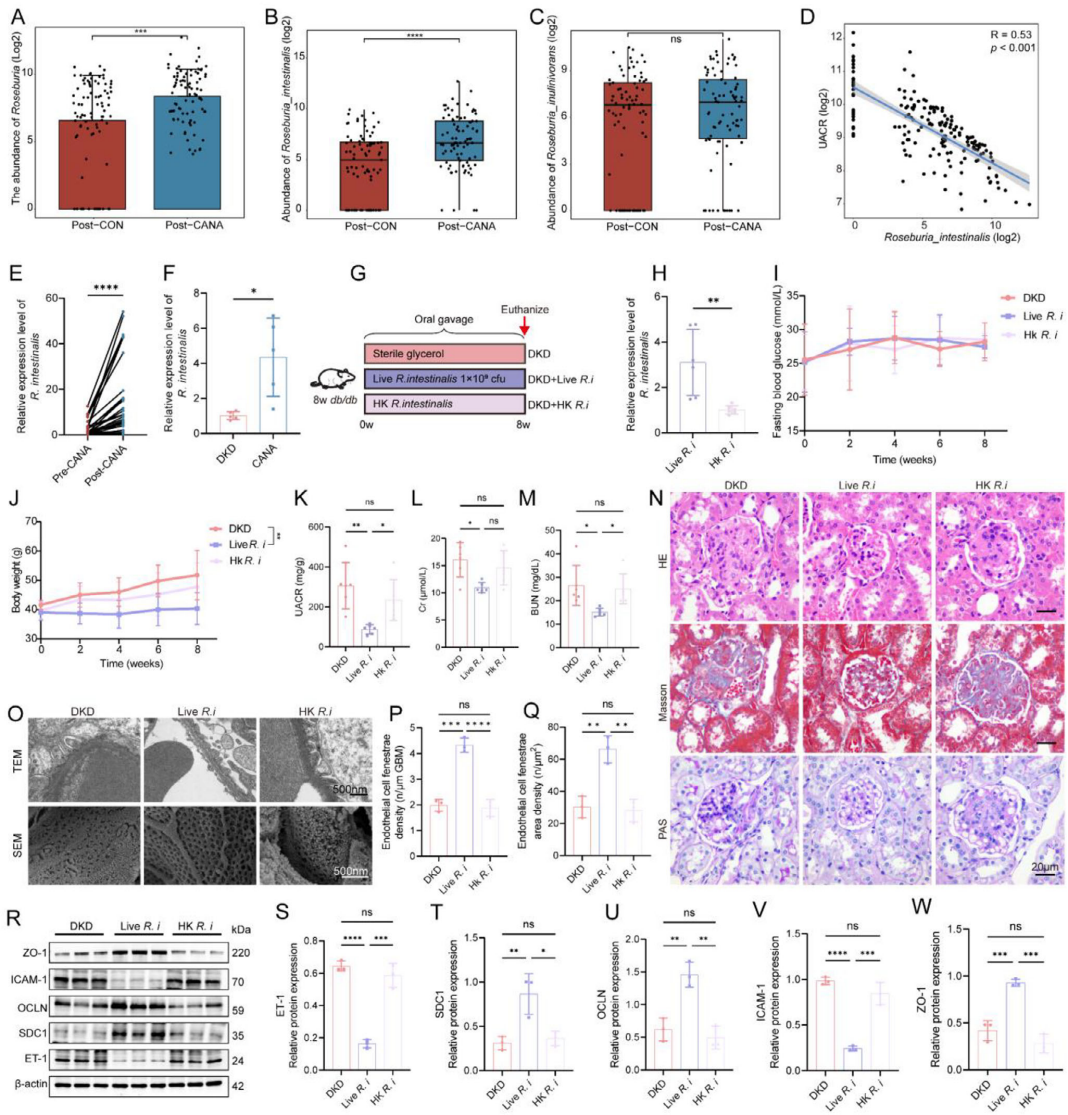
Canagliflozin promotes *R. intestinalis* enrichment, and live *R. intestinalis* ameliorates glomerular endothelial injury in DKD mice. (A) Relative abundance of *Roseburia* based on 16S rRNA sequencing in the CON and CANA groups. (B) Relative abundance of *R. intestinalis* based on 16S rRNA sequencing in the CON and CANA groups. (C) Relative abundance of *R. inulinivorans* based on 16S rRNA sequencing in the CON and CANA groups. (D) Linear regression analysis of the association between the relative abundance of *R. intestinalis* and UACR after treatment. (E,F) Relative abundance of *R. intestinalis* in human feces from pre‐CANA and post‐CANA groups (*n* = 30) (E), and in mouse feces from DKD and CANA groups (*n* = 5) (F). (G) Experimental scheme for *R. intestinalis* intervention. SPF DKD mice were orally administered with sterile glycerol (DKD), live *R. intestinalis* (Live *R.i*), or heat‐killed *R. intestinalis* (HK *R.i*) once a day for 8 weeks (1 × 10^9^ CFU per 0.2 mL per mouse). (H) Relative abundance of *R. intestinalis* in mouse feces from Live *R.i* and HK *R.i* groups (*n* = 6). (I,J) Fasting blood glucose (I) and body weight (J) levels in mice (*n* = 6). (K–M) UACR (K), Cr (L), and BUN (M) levels in mice (*n* = 6). (N) Representative images of H&E, Masson's trichrome, and PAS staining showing DKD‐associated glomerular pathology. Scale bar, 20 µm. (O) Representative TEM and SEM images showing glomerular endothelial fenestration structures. Scale bar, 500 nm. (P) Morphometric quantification of the endothelial cell fenestration density per unit length, performed on TEM images (*n* = 3). (Q) Morphometric quantification of the endothelial cell fenestration area density, performed on SEM images (*n* = 3). (R–W) Representative immunoblotting (R) and quantitative analysis of ET‐1 (S), SDC1 (T), OCLN (U), ICAM‐1 (V), and ZO‐1 (W) proteins in the kidney (*n* = 3). Data are presented as mean ± SD (A–C, F, H‐M, P, Q, S–W). Statistical analysis was performed using the paired two‐tailed Student's test (E), the unpaired two‐tailed Student's test (F, H), the two‐way ANOVA (I, J), and the one‐way ANOVA (K–M, P, Q, S–W). *p < 0.05, **p < 0.01, ***p < 0.001, ****p < 0.0001, ns *p* > 0.05.

As shown in Figures 1F and 3A, canagliflozin increased *Roseburia* abundance, which was strongly correlated with UACR. Two species annotated by 16S rRNA gene sequencing were examined, *Roseburia intestinalis* (*R*. *intestinalis*) and *Roseburia inulinivorans* (*R*. *inulinivorans*), to further clarify which strain of *Roseburia* contributed to this effect. The results indicated that *R. intestinalis*, but not *R*. *inulinivorans*, was significantly increased in the canagliflozin group (Figure 3B,C). This increase remained statistically significant after adjusting for the concomitant use of other classes of antidiabetic therapy (Table S3). In addition, *R. intestinalis* exhibited a strong negative correlation with the UACR (Figure 3D). Quantitative PCR confirmed increased *R. intestinalis* in DKD patients after canagliflozin treatment (Figure 3E), consistent with mouse data (Figure 3F). These results suggest that *R. intestinalis* may play a key role in mediating the effects of canagliflozin on the glomerular endothelium.

DKD mice were supplemented with live *R. intestinalis* (Live *R.i*) or heat‐killed *R. intestinalis* (HK *R.i*) (Figure 3G). Live *R.i* successfully colonized the gut, whereas HK *R.i* did not (Figure 3H). Live *R.i* reduced the body weight without affecting fasting blood glucose levels (Figure 3I,J), and significantly decreased UACR (Figure 3K), creatinine (Figure 3L), and BUN (Figure 3M) levels, whereas HK *R.i* had no effect (Figure 3K–M). Histopathology and electron microscopy images confirmed amelioration of glomerular injury with live *R.i* (Figure 3N–Q). Moreover, kidney protein analysis showed reduced injury markers and increased endothelial integrity markers in the Live *R.i* group (Figure 3R–W). These findings indicated that living *R. intestinalis* could alleviate glomerular endothelial injury, reducing albuminuria.

### 2.5 The Gut Microbial Metabolite Melibiose Alleviates Diabetic Glomerular Endothelial Injury

**FIGURE 4:**
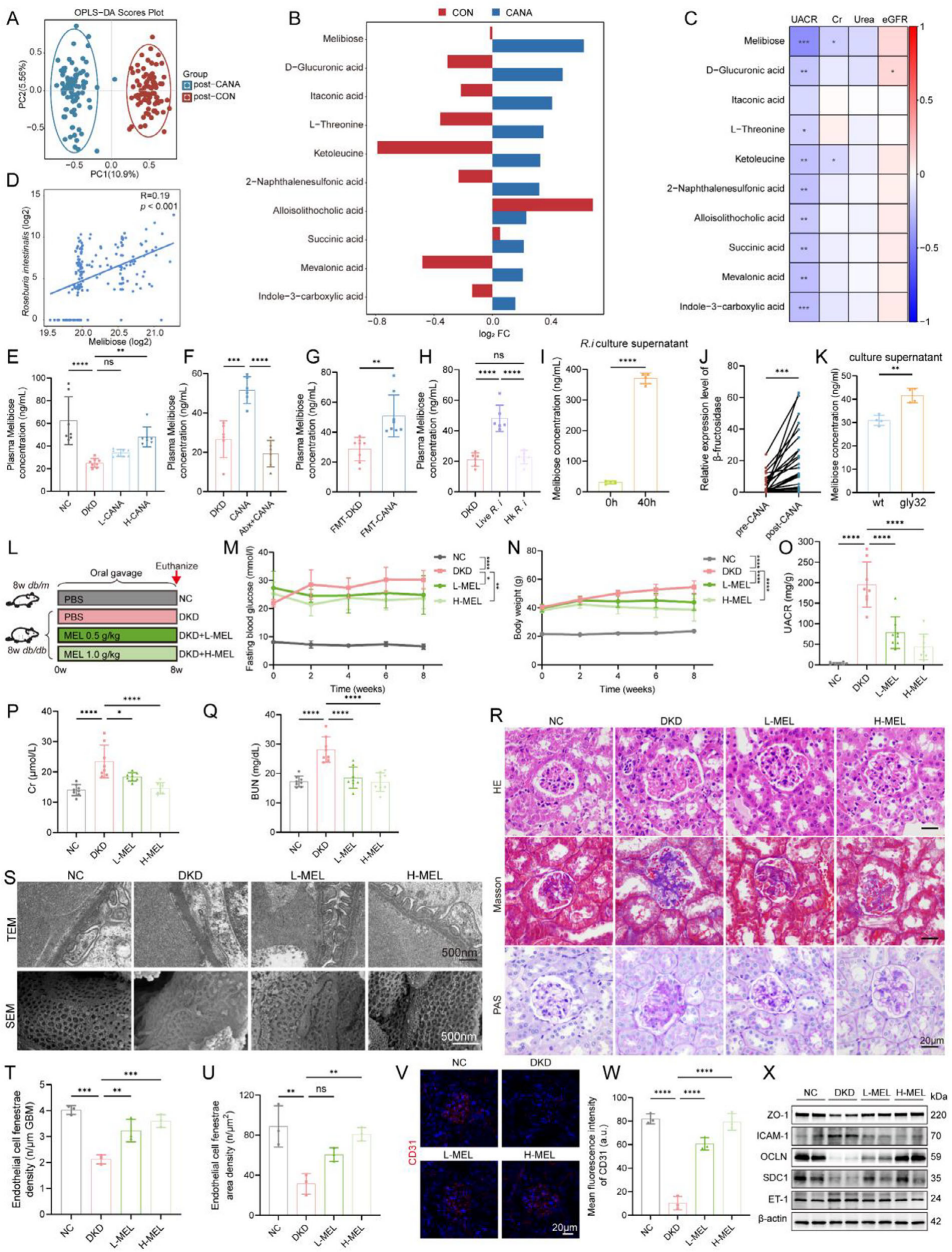
Canagliflozin‐induced gut microbiota metabolite melibiose ameliorates glomerular endothelial cell injury. (A) Orthogonal partial least squares discriminant analysis (OPLS‐DA) showing the metabolites of post‐CON (*n* = 85) and post‐CANA groups (*n* = 85). The post‐CON group is shown in red, and the post‐CANA group is shown in blue. (B) Log2 fold change (log2FC) of the top 10 differential metabolites selected by *p* value, showing within‐group pre‐ and post‐treatment comparisons in CON and CANA groups. Red and blue denote CON and CANA groups, respectively. (C) Heatmap of Pearson's correlations assessing the relationships between the relative levels of selected metabolites and clinical parameters. (D) Linear regression analysis between melibiose and relative abundance of *R. intestinalis* in post‐CON and post‐CANA groups. (E) Melibiose concentrations in mouse plasma after administration of CANA (*n* = 7–8). (F) Melibiose concentrations in mouse plasma of pseudo‐germ‐free mice following CANA administration (*n* = 6). (G) Melibiose concentrations in mouse plasma of pseudo‐germ‐free mice following FMT (*n* = 8). (H) Melibiose concentrations in mouse plasma after administration of *R. intestinalis* (*n* = 6). (I) Melibiose concentrations in the supernatant of *R. intestinalis* cultures (*n* = 4). (J) Relative abundance of *R. intestinalis*‐derived β‐fructosidase in human feces from pre‐CANA and post‐CANA groups (*n* = 30). (K) Melibiose concentrations in the supernatant of wild‐type and gly32‐overexpressing *E. coli* MG1655 strains after 24 h of culture (*n* = 4). (L) Experimental scheme for melibiose intervention. Male DKD mice received melibiose at 0.5 or 1.0 g kg^−1^, or PBS for 8 weeks, and age‐matched non‐DKD control mice were administered PBS. (M,N) Fasting blood glucose (M) and body weight (N) levels in mice (*n* = 8). O‐Q) UACR (O), Cr (P), and BUN (Q) levels in mice (*n* = 8). (R) Representative images of H&E, Masson's trichrome, and PAS staining showing DKD‐associated glomerular pathology. Scale bar, 20 µm. (S) Representative TEM and SEM images showing DKD‐associated glomerular endothelial injury. Scale bar, 500 nm. (T) Morphometric quantification of the endothelial cell fenestration density per unit length, performed on TEM images (*n* = 3). (U) Morphometric quantification of the endothelial cell fenestration area density, performed on SEM images (*n* = 3). (V) Representative immunofluorescence images of glomeruli showing CD31 (red) staining for endothelial cells and DAPI (blue) staining for nuclei in DKD mice. Scale bar, 20 µm. (W) Quantitative analysis of CD31 fluorescence intensity in glomeruli of DKD mice (*n* = 3). (X) Representative immunoblotting of ZO‐1, ICAM‐1, OCLN, SDC1, and ET‐1 proteins in the kidney (*n* = 4). Data are presented as mean ± SD (E–I, K, M–Q, T, U, X). Statistical analysis was performed using the one‐way ANOVA (E–H, O–Q, T, U, X), the unpaired two‐tailed Student's test (I, K), the paired two‐tailed Student's test (J), and the two‐way ANOVA (M, N). *p < 0.05, **p < 0.01, ***p < 0.001, ****p < 0.0001, ns *p* > 0.05.

Given that microbiota‐host crosstalk is largely mediated by bioactive metabolites, widely targeted metabolomics using ultra‐performance liquid chromatography was performed to evaluate plasma metabolic alterations in DKD patients with or without canagliflozin treatment (Figure 1A). Orthogonal partial least squares discriminant analysis (OPLS‐DA) showed clear separation between groups (Figure 4A). Given the central role of dietary and microbially derived carbohydrates in host‐microbiota communication, we specifically focused on this metabolite class in our analysis. Among the top altered metabolites (Figure 4B), the gut microbiota‐derived disaccharide melibiose was significantly increased after canagliflozin treatment and showed the strongest inverse correlation with UACR (Figure 4C). Notably, melibiose levels correlated positively with *R. intestinalis* abundance in the cohort (Figure 4D).

Furthermore, targeted metabolomics in DKD mice confirmed that oral canagliflozin markedly increased plasma melibiose (Figure 4E). This effect was abolished by antibiotic‐mediated microbiota depletion and restored by FMT (Figure 4F,G). Colonization with live *R. intestinalis*, but not PBS or heat‐killed bacteria, further elevated plasma melibiose (Figure 4H), and in vitro cultures of *R. intestinalis* released melibiose above medium background (Figure 4I). These findings prompted us to explore whether *R. intestinalis* is a key bacterial species responsible for melibiose production. The KEGG annotations (map00052, Kanehisa et al., 2025) suggested that β‐fructosidase may be a key enzyme involved in this metabolic process. Notably, the gene expression levels of β‐fructosidase were significantly increased in fecal samples from patients after canagliflozin treatment (Figure 4J). We further validated this result by constructing a recombinant *E. coli* MG1655*^gly32^* strain overexpressing the *R. intestinalis*‐derived β‐fructosidase gene (gly32, WP_006856626.1). Compared with the wild‐type strain, the gly32‐overexpression strain exhibited a significantly higher melibiose production capacity after 24 h of culture (Figure 4K). The above evidence suggested that melibiose might act as a key metabolite derived from *R. intestinalis* that contributes to the renoprotective effects of canagliflozin.

To investigate the renal effects of melibiose in vivo, DKD mice were given daily oral gavage for 8 weeks (Figure 4L). Melibiose treatment did not affect fasting blood glucose (Figure 4M), but reduced body weight gain (Figure 4N), lowered albuminuria (Figure 4O), and improved renal function (Figure 4P,Q). Notably, melibiose markedly alleviated glomerular injury (Figure 4R), restored endothelial fenestration (Figure 4S–U), and preserved glomerular endothelial integrity (Figure 4V,W). Consistently, melibiose upregulated endothelial‐protective proteins and downregulated injury markers (Figure 4X; Figure S4A–E). These benefits were reproduced in an independent diabetic model induced by high‐fat diet (HFD) and streptozotocin (STZ) (Figure S4F–W). In vitro, melibiose (25 or 50 µM) enhanced ZO‐1 and OCLN expression, suppressed ET‐1 and ICAM‐1, and reduced high‐glucose‐induced reactive oxygen species (ROS) accumulation in human glomerular endothelial cells (HGECs), without affecting cell viability (Figure S5A–H). These results demonstrated that melibiose protected glomerular endothelial cells and mitigated endothelial dysfunction in the context of DKD.

### 2.6 Melibiose Directly Binds to GLO1 and Enhances Its Enzymatic Activity

**FIGURE 5:**
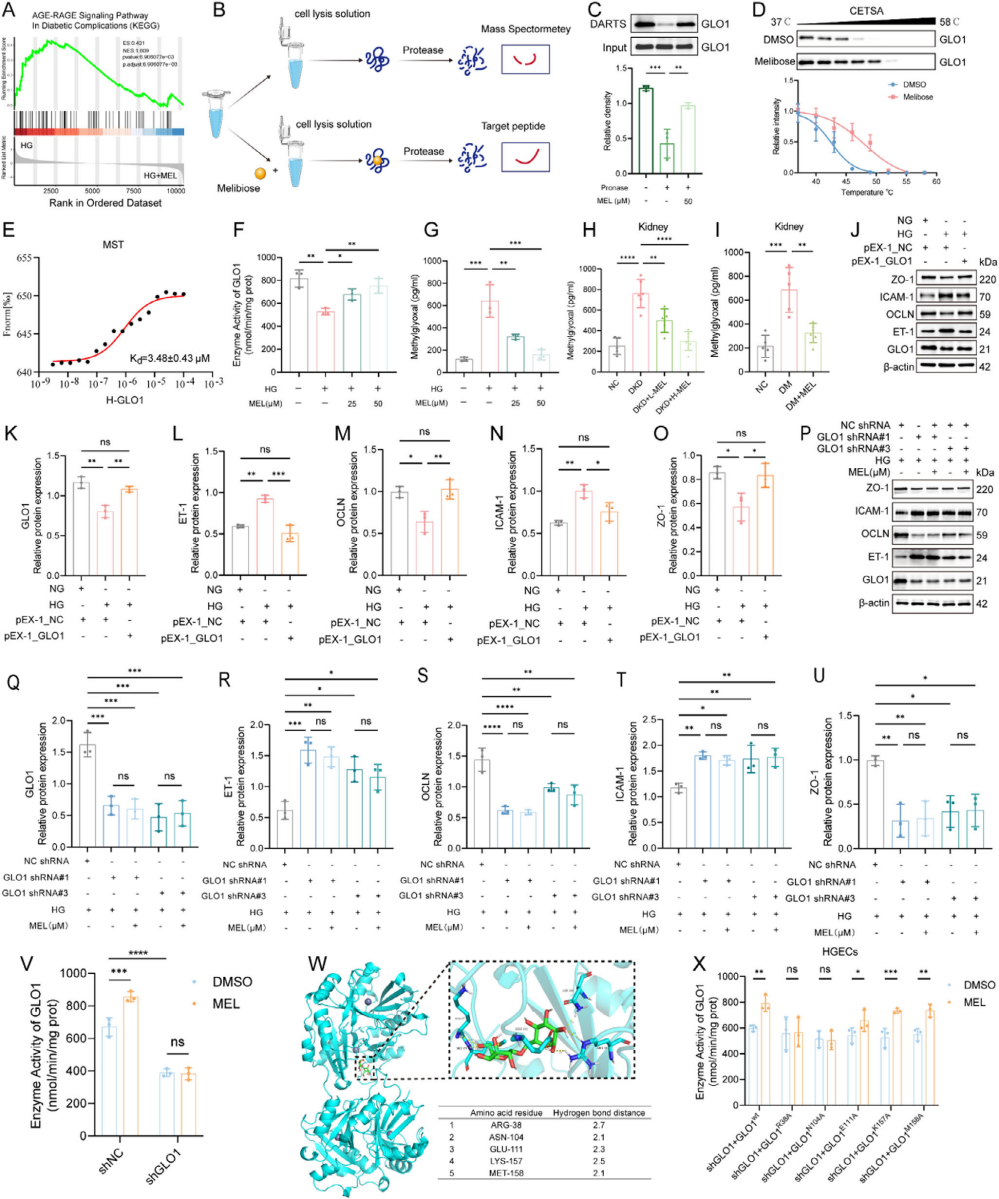
Melibiose directly binds to GLO1 and enhances its enzymatic activity. (A) GSEA of the AGE‐RAGE signaling pathway in diabetic complications based on HGEC transcriptomic data. (B) Schematic diagram showing the workflow for identifying melibiose‐binding proteins using the DARTS approach. (C) Representative immunoblots and quantification from the DARTS assay showing the binding between melibiose and GLO1 (*n* = 3). (D) Representative immunoblots and quantification from the CETSA assay assessing GLO1 stability at different temperatures with or without melibiose treatment (*n* = 3). (E) Microscale thermophoresis (MST) assay showing the direct binding affinity between melibiose and GLO1. (F) GLO1 enzyme activity in HGECs (*n* = 3). (G) Methylglyoxal levels in HGECs (*n* = 3). (H,I) Methylglyoxal levels in mouse kidney (*n* = 5–6). (J–O) Representative immunoblotting (J) and quantitative analysis of GLO1 (K), ET‐1 (L), OCLN (M), ICAM‐1 (N), and ZO‐1 (O) proteins in HGECs following GLO1 overexpression (*n* = 3). P–U) Representative immunoblotting (P) and quantitative analysis of GLO1 (Q), ET‐1 (R), OCLN (S), ICAM‐1 (T), and ZO‐1 (U) proteins in HGECs following GLO1 knockdown (*n* = 3). V) GLO1 enzyme activity in HGECs following GLO1 knockdown (*n* = 3). W) Molecular docking between melibiose and GLO1. Overview of the binding pose and close‐up views of interactions with residues R38, N104, E111, K157, and M158. X) Enzyme activity of GLO1 wild‐type and point mutants re‐expressed in GLO1‐knockdown HGECs (*n* = 3). Data are presented as mean ± SD (C, F–I, K–O, Q–V, X). Statistical analysis was performed using the one‐way ANOVA (C, F–I, K–O, Q–U) and the two‐way ANOVA (V, X). *p < 0.05, **p < 0.01, ***p < 0.001, ****p < 0.0001, ns *p* > 0.05.

Next, to elucidate the mechanism, we performed RNA sequencing on melibiose‐treated HGECs under high‐glucose conditions. Melibiose treatment markedly reduced enrichment of the AGE‐RAGE signaling pathway (Figure 5A), a classical driver of endothelial dysfunction in the diabetic state [21]. We then assessed the levels of TNF‐α and IL‐6, key downstream targets of AGE‐RAGE signaling. Melibiose significantly lowered TNF‐α and IL‐6 levels in diabetic mice (plasma and renal mRNA; Figure S6A–H) and in the culture supernatant of high‐glucose‐treated HGECs (Figure S6I,J). Consistent with this, melibiose also downregulated ET‐1 and ICAM‐1 (Figure 4X; Figure S4R), further supporting inhibition of the AGE‐RAGE axis. To identify the direct targets of melibiose, we applied drug affinity responsive target stability (DARTS) combined with mass spectrometry, which identified 72 candidate proteins (Figure 5B; Figure S6K,L). We prioritized glyoxalase‐1 (GLO1) given its role in detoxifying methylglyoxal, a precursor of AGEs. DARTS (Figure 5C) and the cellular thermal shift assay (CETSA) (Figure 5D) suggested that the increased proteolytic resistance and thermal stability of GLO1 after melibiose treatment. Microscale thermophoresis confirmed direct interaction of melibiose with GLO1, with a dissociation constant (Kd) of 3.48 ± 0.43 µM (Figure 5E).

Importantly, melibiose significantly increased GLO1 enzymatic activity under high‐glucose conditions without altering its protein expression (Figure 5F; Figure S6M). Consistently, methylglyoxal levels were reduced in HGECs (Figure 5G) and renal tissues of diabetic mice (Figure 5H,I). Functionally, GLO1 overexpression alleviated high‐glucose‐induced glomerular endothelial injury (Figure 5J–O). In contrast, GLO1 knockdown significantly decreased its expression and enzymatic activity, exacerbated endothelial injury, and attenuated the protective effect of melibiose (Figure S6N; Figure 5P–V). These findings collectively suggest that melibiose exerts its protective effect by directly binding to GLO1.

Molecular docking predicted that melibiose binds GLO1 via hydrogen bonds with the residues arginine 38 (R38), asparagine 104 (N104), glutamate 111 (E111), lysine 157 (K157), and methionine 158 (M158) (Figure 5W). To validate this, wild‐type or a series of GLO1 mutants (GLO1^R38A^, GLO1^N104A^, GLO1^E111A^, GLO1^K157A^, and GLO1^M158A^) were overexpressed in the GLO1‐knockdown HGECs. Melibiose restored GLO1 activity in cells expressing wild‐type, E111A, K157A, or M158A, but not in R38A or N104A mutants (Figure 5X). Taken together, these findings suggested that melibiose may interact with GLO1 at the R38 and N104 residues, contributing to its protective effect.

In addition, we further investigated whether melibiose exerts protective effects through GLO1 in other glomerular cell types. First, we compared GLO1 expression across these cells and found that its level was higher in glomerular endothelial cells than in podocytes and mesangial cells (Figure S7A,B). Subsequently, under high‐glucose conditions, melibiose treatment similarly alleviated injury in podocytes and mesangial cells. Although this protective effect was less pronounced than that observed in HGECs (Figure S7C–J; Figure S5B–F), it was nevertheless significantly attenuated upon GLO1 knockdown. These findings indicate that GLO1‑mediated protection by melibiose is a shared mechanism across glomerular cell types, with the most prominent effect seen in endothelial cells.

### 2.7 The Protective Effect of Melibiose on the Glomerular Endothelium is Mediated by GLO1

To clarify the role of GLO1 in vivo, we selectively overexpressed GLO1 in glomerular endothelial using an AAV2/9 vector driven by the endothelium‐specific Tie2 promoter (AAV2/9‐Tie2‐GLO1‐mCherry). AAV2/9‐Tie2‐mCherry served as control (Figure S8A). Endothelial GLO1 overexpression did not alter fasting blood glucose, body weight, or creatinine, but reduced albuminuria and modestly lowered BUN (Figure S8B–F). It also decreased plasma TNF‐α and IL‐6 (Figure S8G,H), limited renal methylglyoxal accumulation (Figure S8I), and ameliorated glomerular endothelial injury and dysfunction (Figure S8J–S). These findings suggested GLO1 played a pivotal role in protecting against diabetic glomerular endothelial damage.

**FIGURE 6:**
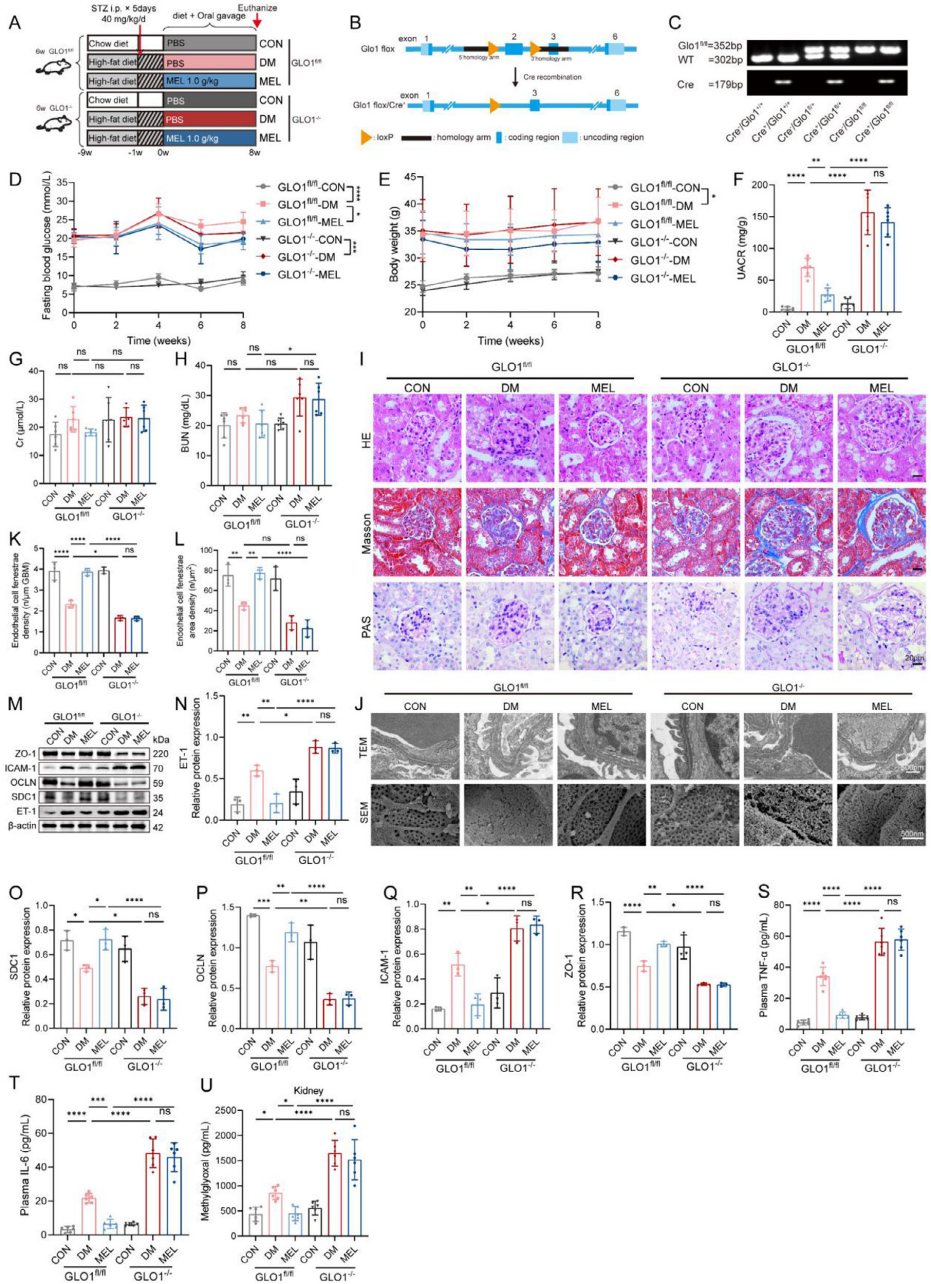
The protective effect of melibiose on the glomerular endothelium is mediated by GLO1. (A) Experimental scheme of the endothelial cell‐specific GLO1 knockout mice. Male GLO1^fl/fl^, cre^−^ (GLO1^fl/fl^) and GLO1^fl/fl^, cre^+^ (GLO1^−/−^) mice were fed either a chow diet (CON) or a high‐fat diet combined with STZ to induce diabetes. Diabetic mice received melibiose (1.0 g kg^−1^, MEL) treatment or PBS (DM). (B) Schematic diagram showing Cre‐mediated recombination between loxP sites flanking exon 2 of the GLO1 allele. Recombination results in the excision of exon 2, generating a GLO1 knockout allele. (C) Genotyping of endothelial cell‐specific GLO1 knockout mice. Representative agarose gel electrophoresis image of PCR products showing six genotypes: Cre^−^ and Cre^+^ combined with GLO1^+/+^, GLO1^fl/+^, and GLO1^fl/fl^ alleles, using genomic DNA extracted from tail tissue of 4‐week‐old mice. (D,E) Fasting blood glucose (D) and body weight (E) levels in mice (*n* = 6). (F–H) UACR (F), Cr (G), and BUN (H) levels in mice (*n* = 6). (I) Representative images of H&E, Masson's trichrome, and PAS staining showing DKD‐associated glomerular pathology. Scale bar, 20 µm. (J) Representative TEM and SEM images showing glomerular endothelial fenestration structures. Scale bar, 500 nm. (K) Morphometric quantification of the endothelial cell fenestration density per unit length, performed on TEM images (*n* = 3). (L) Morphometric quantification of the endothelial cell fenestration area density, performed on SEM images (*n* = 3). (M–R) Representative immunoblotting (M) and quantitative analysis of ET‐1 (N), SDC1 (O), OCLN (P), ICAM‐1 (Q), and ZO‐1 (R) proteins in the kidney (*n* = 3). (S–U) Plasma TNF‐α (S), plasma IL‐6 (T), and renal methylglyoxal (U) levels in mice (*n* = 6). Data are presented as mean ± SD (D–H, K, L, N–U). Statistical analysis was performed using the one‐way ANOVA (F–H, K, L, N–U) and the two‐way ANOVA (D, E). *p < 0.05, **p < 0.01, ***p < 0.001, ****p < 0.0001, ns *p* > 0.05.

Next, to further investigate the role of GLO1 in mediating the protective effects of melibiose, endothelial‐specific GLO1 knockout (GLO1^−/−^) mice were generated (Figure 6A–C). GLO1 deletion had no significant effect on fasting blood glucose levels, body weight, creatinine or BUN levels, but significantly increased albuminuria (Figure 6D–H) and aggravated glomerular endothelial injury in diabetic mice (Figure 6I–R). Notably, compared with GLO1*^fl/fl^* mice, melibiose treatment failed to reduce albuminuria (Figure 6F) or decrease the plasma levels of TNF‐α (Figure 6S), IL‐6 (Figure 6T), and renal methylglyoxal (Figure 6U) in GLO1^−/−^ mice. In addition, the protective effects of melibiose on glomerular endothelial injury were abolished in the absence of GLO1 (Figure 6I–U).

To elucidate the role of renal GLO1 in mediating the effects of melibiose, we used AAV2/9‐TIE‐mir30‐mCherry to specifically knock down GLO1 in glomerular endothelial cells of diabetic mice, with the control virus as a comparator (Figure S9A). The results closely resembled those observed in GLO1^−/−^ mice (Figure S9B–S). Therefore, these findings support the notion that the protective effect of melibiose on the glomerular endothelium is dependent on GLO1 within glomerular endothelial cells.

### 2.8 Potential Therapeutic Efficacy of Melibiose in DKD Patients

**FIGURE 7:**
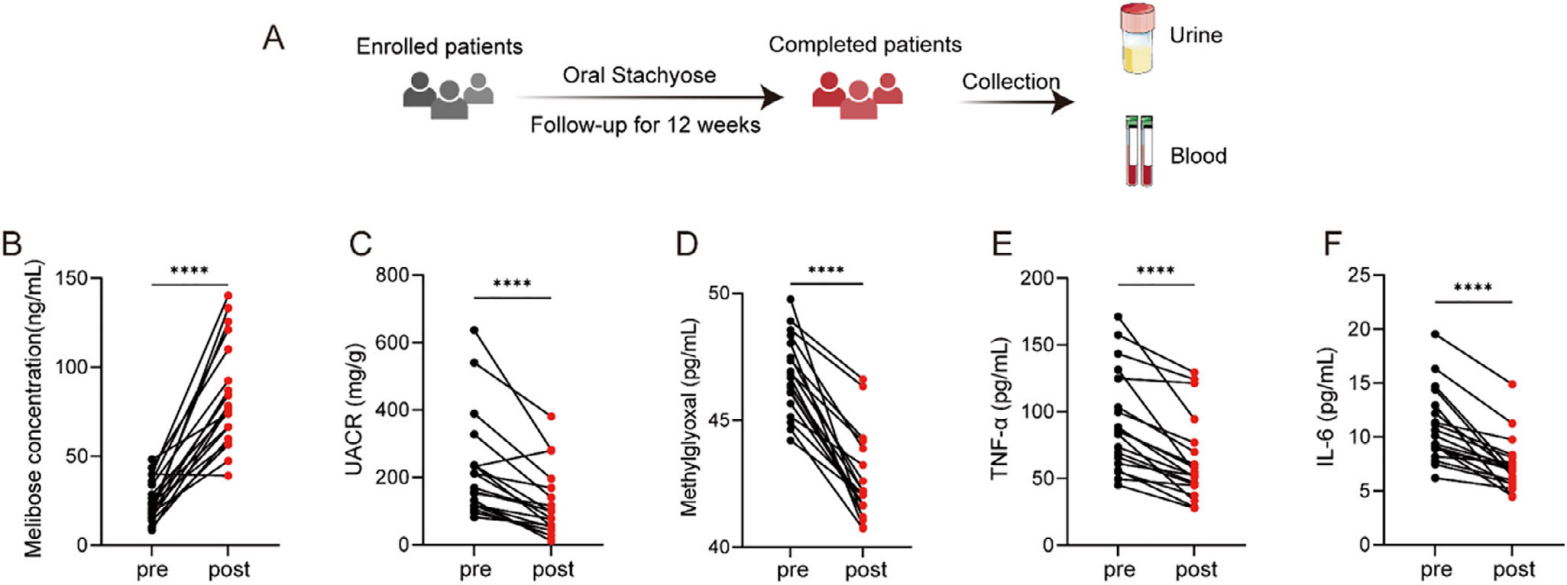
Melibiose precursor elevates plasma melibiose and attenuates albuminuria in DKD patients. (A) Clinical study flow chart of DKD patients receiving stachyose (*n* = 19). Blood and urine samples were collected at baseline and post‐intervention. (B) Plasma melibiose levels in stachyose‐treated patients (*n* = 19). (C) UACR levels in stachyose‐treated patients (*n* = 19). (D–F) Plasma methylglyoxal (D), TNF‐α (E) and plasma IL‐6 (F) levels in stachyose‐treated patients (*n* = 19). Each dot represents a single sample, black and red indicate pre‐ and post‐treatment, respectively (B–F). Statistical analysis was performed using the paired two‐tailed Student's test (B, D–F) and Wilcoxon signed‐rank test (C). ****p < 0.0001.

Finally, we performed a 12‐week pilot clinical study to evaluate the efficacy of melibiose in the treatment of patients with DKD (Figure 7A; Figure S10). Given the unavailability of food‐grade melibiose, we used stachyose, an FDA‐recognized generally recognized as safe (GRAS) precursor, for dietary supplementation (5 g twice daily). Nineteen DKD patients (baseline UACR 30–1000 mg g^−1^) completed the study with no changes to background therapy. Stachyose significantly raised plasma melibiose (Figure 7B) and reduced UACR (Figure 7C), circulating methylglyoxal (Figure 7D), TNF‐α (Figure 7E), and IL‐6 (Figure 7F). Modest decreases in BMI, HbA1c, and uric acid were also observed (Table S4). Findings were corroborated in DKD model mice (Figure S11A–K). Together, these findings suggest that melibiose has potential therapeutic value for patients with DKD.

## 3 Discussion

In this study, we investigated the impacts of canagliflozin on the gut microbiota and metabolic profiles of patients with DKD. Integrated microbiome and metabolome analyses revealed that canagliflozin reshaped the gut microbial composition, with *R. intestinalis* emerging as a potential microbial mediator of its antiproteinuric effects. Melibiose, a key metabolite produced by *R. intestinalis*, contributed to reducing albuminuria. Notably, melibiose exerted significant antiproteinuric effects in both a DKD model and a HFD combined with STZ‐induced diabetes model. Mechanistically, melibiose directly bound to GLO1, enhanced its enzymatic activity, and subsequently downregulated the AGE‐RAGE signaling pathway. Moreover, a pilot clinical study showed that supplementation with the precursor of melibiose alleviated albuminuria in patients with early‐stage DKD. Collectively, our findings highlight the therapeutic potential of targeting the gut‐kidney axis and microbe‐metabolite interactions in the management of DKD.

Previous investigations into the renoprotective mechanisms of SGLT2 inhibitors have focused primarily on direct renal actions, mainly due to their high selectivity for SGLT2 [22, 23]. However, canagliflozin inhibits intestinal SGLT1 by approximately 40%–60% [24], allowing a greater proportion of carbohydrates to escape absorption and reach the cecum, a phenomenon confirmed in both human and mouse studies [9, 25]. Although previous studies have reported the effects of canagliflozin on the gut microbiota in animal models of type 2 diabetes and hypertension‐induced kidney injury [10, 26, 27], evidence from studies in patients with DKD remains limited. Therefore, this prospective controlled cohort study systematically assessed the effects of canagliflozin on the gut microbiota in DKD patients. In our research, canagliflozin treatment significantly reshaped the gut microbial composition in DKD patients, notably increasing the F/B ratio. This finding contrasts with previous research on the impact of canagliflozin on the gut microbiota in patients with type 2 diabetes mellitus [10]. The underlying reason likely lies in the fundamental differences in baseline gut microbiota between type 2 diabetes mellitus and DKD, which represent distinct stages of disease progression. Therefore, assessing drug‐induced microbial shifts requires careful consideration of the specific pathological context. Supporting this view, a meta‐analysis including 578 DKD patients and 444 healthy controls revealed that DKD itself is characterized by increased Bacteroidetes abundance and reduced Firmicutes abundance [28]. Thus, the elevated F/B ratio observed after canagliflozin treatment in DKD patients may reflect a targeted correction of this disease‐specific dysbiosis, rather than representing an inconsistency with findings in type 2 diabetes mellitus populations. Notably, the genus *Roseburia* has been consistently reported to be depleted in patients with DKD and is closely associated with the renal function decline [28, 29]. We observed a significant restoration of *Roseburia* abundance following canagliflozin treatment, which was strongly correlated with reductions in the UACR. More importantly, our results identified *R. intestinalis*, a potential probiotic that is markedly reduced in DKD patients according to metagenomic evidence [30], as a key microbial mediator of the antiproteinuric effect of canagliflozin. Studies have shown that *R. intestinalis* grows robustly in the presence of carbohydrates [31], which may partly explain the observed increase in its abundance following canagliflozin treatment.

Although GECs do not express SGLT2 [32], we were surprised to observe that canagliflozin treatment significantly alleviated glomerular endothelial injury in DKD model mice. This protective effect was evidenced by an improved endothelial morphology, restored fenestration, increased glycocalyx and tight junction protein expression, and reduced inflammation and injury. Importantly, the *db/db* mouse model has been well‐established to consistently recapitulate the glomerular endothelial injury observed in human DKD, providing a reliable tool for investigating this pathological process [33, 34]. Previous studies have reported similar endothelial protective effects of SGLT2 inhibitors, potentially through mechanisms such as the suppression of endothelial‐to‐mesenchymal transition [35], a reduction in endothelial apoptosis, and the restoration of cellular function [32]. However, the underlying mechanisms remain incompletely understood. Our study proposed that the gut‐kidney axis may play a critical role in mediating the protective effects of canagliflozin on glomerular endothelial cells. Given that these cells are directly exposed to circulating metabolites, this finding provides a physiological basis for gut microbiota‐derived metabolites to exert endothelial‐protective effects. Moreover, glomerular endothelial dysfunction has been recognized as an early event in DKD and may precede the onset of microalbuminuria [36, 37]. Importantly, studies in DKD‐susceptible mouse models further suggest that glomerular endothelial injury may occur before overt podocyte injury or depletion [38, 39]. Early improvements in endothelial structure and function may offer long‐term renal benefits by modulating the crosstalk between GECs and podocytes [40, 41]. This result provides a mechanistic explanation for the potent antiproteinuric effect observed in our study, which was mediated by gut microbiota‐derived metabolites that ameliorated glomerular endothelial injury, and highlights a previously underappreciated role for the gut‐kidney axis in the therapeutic action of canagliflozin.

In our study, melibiose emerged as a key metabolite linking *R. intestinalis* to glomerular endothelial protection, thereby mediating the antiproteinuric effects of canagliflozin. Notably, the oral administration of live *R. intestinalis*, but not heat‐killed bacteria, significantly increased melibiose levels in vivo. In vitro culture experiments further suggested that *R. intestinalis* is capable of producing melibiose. It is important to note that melibiose in the host intestine is primarily hydrolyzed from dietary sources [42]. Correspondingly, the culture medium for *R. intestinalis* is rich in dietary components such as peptone, beef extract, and yeast extract, which likely serve as sources of oligosaccharide precursors. Previous studies reported that melibiose is a gut microbiota‐derived metabolite [43] and that *R. intestinalis* exhibits strong metabolic activity toward melibiose precursors but has a limited ability to degrade melibiose itself [44], which is consistent with our current findings. Moreover, canagliflozin treatment significantly increased melibiose levels in conventional DKD model mice, whereas this effect was abolished in microbiota‐depleted mice. Taken together, these data indicate that the presence of viable gut microbes, particularly *R. intestinalis*, is essential for the canagliflozin‐induced increase in melibiose levels, highlighting the potential role of the gut‐kidney axis in mediating the renoprotective effects of canagliflozin. Importantly, we observed that melibiose itself did not affect blood glucose levels in the DKD model, suggesting that the antiproteinuric effects of canagliflozin may be partly independent of glucose lowering. Nevertheless, we recognize that the primary mechanism of canagliflozin, inhibition of SGLT1/2 and subsequent increase in carbohydrate delivery to the gut, serves as the initiating event for this microbe‐metabolite cascade. Thus, while the renal protective effects appear to be distinct, at least in part, from glucose‐lowering, the overall protective process remains rooted in a glucose‐centric pharmacological action. Additionally, the possibility that canagliflozin may also modulate other microbial taxa or stimulate the production of additional beneficial metabolites cannot be excluded and needs to be investigated in the future.

Melibiose is a disaccharide composed of galactose and glucose linked by an α‐1,6‐glycosidic bond. In our study, melibiose did not affect blood glucose levels in DKD mice, supporting our hypothesis that canagliflozin exerts antiproteinuric effects through melibiose independent of its glucose‐lowering activity. Previous studies have shown that melibiose and its precursor, stachyose, may be upregulated during acute kidney injury as part of an endogenous protective mechanism [45]. Our results suggested that melibiose also acts as a renoprotective metabolite, with reduced levels in DKD patients that are restored following canagliflozin treatment. The differences in changes in melibiose levels may be influenced by the disease duration. Melibiose has previously been reported to exert neuroprotective effects by suppressing oxidative stress, attenuating inflammation, and enhancing autophagic flux [19, 20]. However, its role in kidney disease has not been well characterized. Here, we revealed for the first time that melibiose contributes to renal protection in individuals with DKD by directly binding to GLO1, increasing its enzymatic activity and thereby reducing the accumulation of methylglyoxal, a key precursor of AGEs, indirectly downregulating the AGE‐RAGE signaling pathway. GLO1 is a central enzyme in the glyoxalase system that is responsible for detoxifying methylglyoxal, and its protective effect on diabetic microvascular complications has been well established [46, 47]. Furthermore, previous studies have shown that pharmacological inducers of GLO1 can alleviate DKD pathology [48]. Consistent with these findings, our findings support melibiose as a novel GLO1 inducer with renoprotective potential. Moreover, the AGE‐RAGE signaling pathway plays a pivotal role in the progression of DKD [1, 49]. Our results showed that melibiose significantly reduced the expression of key downstream targets of the AGE‐RAGE pathway, including ET‐1, ICAM‐1, TNF‐α, and IL‐6, indicating a suppressive effect of melibiose on this signaling cascade. This inhibition may alleviate glomerular endothelial injury under diabetic conditions and help slow disease progression.

Previous preclinical studies have shown that AGE inhibitors and RAGE‐blocking antibodies can ameliorate DKD pathology [50, 51]. While these findings are consistent with our results, the underlying mechanisms differ. In our study, melibiose indirectly inhibited the AGE‐RAGE signaling pathway, seemingly by increasing GLO1 activity to reduce AGE formation at its source, thus mediating the renoprotective effect. However, clinical trials confirming the end results of targeting the AGE‐RAGE axis in DKD patients are still lacking [52]. Due to the limited availability of food‐grade melibiose, we used its precursor, stachyose, as a substitute to preliminarily evaluate its clinical efficacy. Notably, in our study, a pilot clinical trial showed that stachyose intervention led to an approximately 40% reduction in UACR in patients with early‐stage DKD, indirectly suggesting a potential renoprotective effect of melibiose. Although melibiose was not directly administered, these findings provide valuable insights and a rationale for future clinical investigations. In addition, given that melibiose is a gut microbiota‐derived metabolite, its protective effects may involve multiple cellular and molecular mechanisms beyond binding to GLO1. Thus, it may influence not only glomerular endothelial cells but also other cellular functions, and these multifaceted effects may together be necessary for its protective effects on albuminuria and warrant investigation in future studies.

Despite the significant findings of our study, several limitations should be acknowledged. One limitation is that the current clinical trial evaluating the oral administration of the melibiose precursor was conducted as an open‐label, uncontrolled pilot study. While the observed effects are promising, they could be influenced by other confounding factors, which limits the ability to fully assess therapeutic efficacy. Future studies with larger sample sizes and randomized controlled designs are warranted to further validate the clinical value of these findings. Another is that while we focused on the key metabolite melibiose produced by *R. intestinalis*, other metabolites may also contribute to the effects of canagliflozin on the gut‐kidney axis, which require further exploration. Finally, this study was limited by the inability to conduct genetic knockout in *R. intestinalis*. The development of suitable genetic tools remains an important objective for subsequent research.

In conclusion, by conducting a holistic investigation, our study reveals a pivotal role of the canagliflozin‐modulated gut microbiota and implicates such interactions in the intervention of DKD. Notably, *R. intestinalis*‐derived melibiose increases the activity of its protein target GLO1, thereby inhibiting the AGE‐RAGE signaling pathway, alleviating glomerular endothelial damage, and ultimately reducing albuminuria in individuals with DKD. Importantly, we further confirmed that the oral administration of the melibiose precursor also has therapeutic potential against albuminuria in patients with early‐stage DKD, which underscores the clinical translational value of this study. Collectively, these findings deepen our understanding of the gut‐kidney in DKD and provide a novel potential therapeutic avenue for mitigating albuminuria in clinical practice.

## 4 Experimental Methods

### 4.1 Experimental Model and Subject Details

#### 4.1.1 Human Study

All the human studies were approved by the Ethics Committee of the First Affiliated Hospital of Zhengzhou University and adhered to the principles of the Declaration of Helsinki. All relevant ethical regulations were followed during the study. Written informed consent was obtained from all participants. Both clinical investigations were registered at the Chinese Clinical Trial Registry (ChiCTR2200062178 and ChiCTR2400089400).

#### 4.1.2 Human Cohort 1

For the canagliflozin cohort, 200 individuals who met all the following eligibility criteria were recruited from the First Affiliated Hospital of Zhengzhou University, the Second Affiliated Hospital of Zhengzhou University, the Fifth Affiliated Hospital of Zhengzhou University, Zhengzhou Central Hospital, the Seventh People's Hospital of Zhengzhou, Hebi Coal Industry Group General Hospital, Xinxiang Central Hospital, and Zhumadian Central Hospital. The inclusion criteria were as follows: (i) age ≥ 30 years with a diagnosis of type 2 diabetes; (ii) a glycated hemoglobin level of 6.5%–12.0%; and (iii) the presence of chronic kidney disease (CKD), defined as an estimated glomerular filtration rate of ≥30–<90 mL per minute per 1.73 m^2^ of body surface area, (iv) along with albuminuria, as indicated by a UACR ranging from >300 to 5000 mg g^−1^. The subjects were excluded if they (i) had a diagnosis of type 1 diabetes mellitus or a history of diabetic ketoacidosis; (ii) had a history of hereditary glucose‐galactose malabsorption or primary renal glycosuria; (iii) had severe cardiovascular or cerebrovascular disease; (iv) had a diagnosis of kidney disease requiring immunosuppressive therapy; (v) had a history of kidney transplantation or were undergoing dialysis; or (vi) had a known allergy or intolerance to SGLT2 inhibitors. All participants had received stable doses of angiotensin‐converting enzyme inhibitors (ACEIs) or angiotensin receptor blockers (ARBs) for at least 8 weeks prior to enrollment. None had been treated with SGLT2 inhibitors or glucagon‐like peptide‐1 (GLP‐1) receptor agonists before enrollment. In addition, all participants had been on a stable antidiabetic regimen for at least 4 weeks before study entry. Patients were randomly assigned in a 1:1 ratio to either the treatment group or the control group (100 patients per group) using a computer‐generated random sequence. After excluding four participants who discontinued the intervention, eighteen who were lost to follow‐up, and eight who used antibiotics during the sample collection period, a total of 170 participants were included in the final analysis (CON, *n* = 85; CANA, *n* = 85). Plasma, urine, and fecal samples were collected at baseline and at the end of the study.

#### 4.1.3 Human Cohort 2

For the stachyose supplement study, 20 participants were recruited from the First Affiliated Hospital of Zhengzhou University when they met the following criteria: (i) diagnosed with type 2 diabetes mellitus for at least one year, aged 18–80 years, with a glycated hemoglobin level ≤9% and a body mass index ≤30 kg m^−2^; (ii) clinically diagnosed with CKD and persistent albuminuria for at least six months, defined as a UACR>30 to ≤1000 mg g^−1^ and an eGFR ≥ 60 mL min^−1^ 1.73 m^−2^, without evidence of nondiabetic kidney disease; (iii) receiving a stable treatment regimen for at least 4 weeks, including a stable dose of an ACEI or ARB, and not receiving SGLT2 inhibitors, GLP‐1 receptor agonists, or finerenone; (iv) no history of acute or chronic hepatitis with increased transaminase levels, heart failure, malignant, dialysis, or organ transplantation; (v) no uncontrolled hypertension or a blood potassium level >5.5 mmol L^−1^; (vi) not pregnant, planning to become pregnant, or currently lactating; and (vii) willing to participate in the study and demonstrated good adherence to medical recommendations. Stachyose (5 g; Zangling Industrial Co., Ltd., Luoyang, China) was administered twice daily, while all other treatments remained unchanged. HbA1c levels, the UACR, renal function, and circulating lipid levels were evaluated after 12 weeks of follow‐up. Plasma and urine samples were collected from patients recruited at the beginning and end of the study. One patient discontinued participation in the study before completing the intervention.

#### 4.1.4 Mouse Model

All the animal studies were approved by the Institutional Animal Care and Use Committee of the Henan Academy of Medical and Pharmaceutical Sciences (Approval No. 2023‐yyy‐047) and were conducted in strict accordance with established ethical guidelines for animal research. All the mice were maintained in a specific pathogen‐free (SPF) environment on a 12 h light/12 h dark cycle at 22°C ± 2°C and 55% ± 5% humidity with free access to food and water. Adult male BKS‐*db/db*, BKS‐*db/m*, and C57BL/6J mice aged 6–8 weeks were purchased from GemPharmatech Co., Ltd. (Jiangsu, China). Glo1‐Flox mice (Cat. No. NM‐CKO‐200322) were purchased from Shanghai Model Organisms Center, Inc. (Shanghai, China), and Tek‐Cre mice (Cat. No. YDS0126) were purchased from Shulaibao Biotechnology Co., Ltd. (Wuhan, China).

Before the start of the experiment, fasting blood glucose (FBG) levels and the urinary albumin‐to‐creatinine ratio (UACR) were measured in all BKS‐*db/db* mice. Mice with an FBG concentration ≥16.7 mmol L^−1^ and a UACR ≥ 30 mg g^−1^ were considered to be a successful DKD model and were used for subsequent experiments. Mice on the C57BL/6J background were fed a high‐fat diet (HFD) (60% kcal from fat) throughout the study and received intraperitoneal injections of streptozotocin (40 mg kg^−1^ day^−1^) for five consecutive days after 8 weeks of feeding to induce diabetes. The HFD/STZ protocol was also applied in genetically modified GLO1 mice to establish diabetic models. Mice with an FBG level ≥ 16.7 mmol L^−1^ were considered successfully modeled. Additionally, all mice were monitored daily for signs of discomfort, and body weight and blood glucose levels were regularly checked to ensure prompt intervention if necessary. Prior to euthanasia, all mice were deeply anesthetized to ensure a painless procedure.

#### 4.1.5 Bacterial Culture

*Roseburia intestinalis* (*R. intestinalis*, JCM 31262, Bio105771) was cultured in reinforced clostridial medium (RCM) at 37°C in an anaerobic workstation (Don Whitley Scientific, UK). Uninoculated RCM medium served as a blank control. For the in vitro targeted metabolomic analysis, culture supernatants were collected after 40 h fermentation. For the in vivo colonization experiment, *R. intestinalis* in the logarithmic growth phase was collected by centrifugation at 4000 rpm for 10 min at 4°C. The bacterial pellet was washed and resuspended in sterile phosphate‐buffered saline (PBS), and the optical density at 600 nm (OD_600_) was measured using a spectrophotometer. OD_600_ values between 1.0 and 1.2 corresponded to a bacterial concentration of approximately 10^8^–10^9^ CFUs mL^−1^. The final bacterial suspension was mixed with sterile glycerol to a final concentration of 20%.

Wild‐type *E. coli* MG1655 and *E. coli* MG1655 overexpressing the *R. intestinalis*‐derived β‐fructosidase gene (gly32, WP_006856626.1) were cultured in PBS supplemented with stachyose as the disaccharide substrate. Cultures were incubated at 37°C for 24 h. After incubation, the supernatants were collected by centrifugation and subjected to mass spectrometry analysis.

#### 4.1.6 Cell Lines and Culture Conditions

Human glomerular endothelial cells (HGECs, ScienCell Research Laboratories, Carlsbad, USA) were cultured in RPMI‐1640 medium (Servicebio, Wuhan, China) (5.6 mmol L^−1^ glucose) supplemented with 10% fetal bovine serum (FBS, Shuangru Biotech, Suzhou, China). SV40 MES 13 mouse mesangial cells (National Collection of Authenticated Cell Cultures, Shanghai, China) were maintained in DMEM/F12 medium (Procell, Wuhan, China) (5.6 mmol L^−1^ glucose) supplemented with 10% FBS. Both HGECs and mesangial cells were maintained in a 37°C incubator with 5% CO_2_.

Mouse podocyte clone‐5 (MPC‐5; National Collection of Authenticated Cell Cultures, Shanghai, China) cells were cultured in a two‐stage protocol. For proliferation, cells were grown in DMEM (Procell, Wuhan, China) with 5.6 mmol L^−1^ glucose, 10% FBS, and 100 U mL^−1^ recombinant mouse interferon‐gamma (IFN‐γ; Procell, Wuhan, China) at 33°C under 5% CO_2_. To induce differentiation, cells at 80% confluence were switched to the same medium without IFN‐γ and maintained at 37°C for 10 days. Medium for all cell types was refreshed every 2–3 days, and cells were subcultured upon reaching approximately 80% confluence.

Melibiose (Cat. T2914, Taoshu Biotechnology, Shanghai, China) was dissolved in DMSO to form a stock solution and diluted with serum‐free medium to working concentrations of 25 and 50 µM. The concentration range for the cell experiments was determined based on preliminary cytotoxicity assays.

For the experiments, the cells were plated on six‐well plates at a density of 1 × 10^6^ cells/well and allowed to adhere overnight. The cells were incubated with melibiose and high glucose (30 mmol L^−1^) for 48 h. The cells were then collected for further assays.

### 4.2 Method Details

#### 4.2.1 Study Design

##### 4.2.1.1 Human Cohort 1

This study was a prospective, multicenter, interventional, randomized controlled trial. Patient recruitment was conducted between August 2022 and January 2023, followed by a 26‐week intervention. Patients were recruited from the First Affiliated Hospital of Zhengzhou University (the leading center) and seven other clinical centers in China. Participants were randomized at a 1:1 ratio to receive either canagliflozin (Invokana, 100 mg once daily with breakfast; Janssen, USA) or glucose‐lowering medications adjusted according to blood glucose levels, excluding any SGLT2 inhibitors. All participants were advised to maintain their usual dietary patterns and prestudy medical regimens, including antihypertensive and lipid‐lowering therapies. Use of antibiotics or probiotics during the sample collection period was removed from the final analysis.

The participants were followed regularly through telephone interviews and in‐person clinic visits. Telephone follow‐ups were conducted every 4 weeks to monitor medication adherence, dietary and physical activity habits, and adverse events. An in‐person visit was conducted at week 26 at the Endocrinology Clinic of the First Affiliated Hospital of Zhengzhou University, during which blood, urine, and stool samples were collected, aliquoted, and stored for subsequent analyses. The UACR was uniformly measured for all participants at the First Affiliated Hospital of Zhengzhou University.

##### 4.2.1.2 Human Cohort 2

This study was a 12‐week, prospective clinical trial. Twenty eligible participants were enrolled in August 2024 at the Endocrinology Department of the First Affiliated Hospital of Zhengzhou University. All patients had received stable treatment regimens for at least 4 weeks, including lifestyle interventions, glucose control, and blood pressure management. Patients received oral stachyose (5 g, twice daily) as a supplement during the study. The original treatments remained unchanged.

The participants were assessed every 4 weeks to monitor medication adherence and adverse events. A face‐to‐face follow‐up was conducted at week 12. Blood and urine samples were collected at both baseline and week 12 and were stored for subsequent analysis. Adherence to stachyose supplementation was assessed by counting the number of empty stachyose packaging bags returned by the participants at each visit.

#### 4.2.2 Clinical Measurements and Biospecimen Collection

Systolic and diastolic blood pressures were measured using an automated electronic sphygmomanometer (Omron HBP‐1300, Omron Healthcare, Japan) after participants had rested in a seated position for at least 5 min.

Body weight and height were measured in the morning after an overnight fast and after voiding urine and stool. Participants wore light clothing and no shoes during the measurements. Body mass index (BMI) was calculated as weight in kilograms divided by height in meters squared (kg m^−2^).

Fasting venous blood samples were collected from participants between 8:00 and 12:00 a.m. These samples were used to measure glycated hemoglobin (HbA1c), urea, creatinine (Cr), uric acid (UA), total cholesterol (TC), triglyceride (TG), high‐density lipoprotein cholesterol (HDL‐C), and low‐density lipoprotein cholesterol (LDL‐C) levels using a Cobas 8000 analyzer (Roche Diagnostics, Mannheim, Germany). Plasma levels of tumor necrosis factor‐α (TNF‐α) and interleukin‐6 (IL‐6) were measured using a Human ELISA Kit (TNF‐α, Cat.JL10208; IL‐6, Cat. JL14113) from JonlnBio Industrial Co., Ltd. (Shanghai, China). Plasma methylglyoxal levels were measured using a Human methylglyoxal ELISA kit (Cat. MM‐60917H1, Jiangsu Meimian Industrial Co., Ltd., Yancheng, China).

Midstream urine samples were collected from the first morning void of each participant. The UACR was measured using a DIRUI CS‐400 analyzer (DIRUI Industrial Co., Ltd., China).

Fresh stool was collected into a clean container, avoiding contamination with urine or toilet surfaces. The central portion of the stool segment was homogenized, and then approximately 2–3 g of the sample was collected into sterile tubes. Samples were immediately snap‐frozen in liquid nitrogen for 15 min and stored at −80°C until DNA extraction.

#### 4.2.3 16S rRNA Sequencing

Frozen fecal samples from study participants and mice were thawed and thoroughly homogenized with extraction lysis buffer. Total genomic DNA was extracted using the OMEGA Soil DNA Kit (Cat. M5635‐02; Omega Bio‐Tek, Norcross, USA). DNA quality and concentration were assessed via agarose gel electrophoresis and a Nanodrop spectrophotometer (Thermo Fisher Scientific, USA). The V3‐V4 regions of the bacterial 16S rRNA genes were amplified by PCR using region‐specific primers. The primers used are listed in Table S5. The PCR products were confirmed by agarose gel electrophoresis, purified, and quantified. Sequencing libraries were constructed using the Illumina TruSeq Nano DNA LT Library Prep Kit (Illumina, USA). After quality control and quantification, the libraries were sequenced on the Illumina NovaSeq 6000 platform using the NovaSeq 6000 SP Reagent Kit (500 cycles) for paired‐end 2 × 250 bp sequencing.

#### 4.2.4 Large‐Scale Targeted Metabolomics Analysis

##### 4.2.4.1 Sample Pre‐Treatment

For the plasma samples, 100 µL aliquots were thawed at 4°C and mixed with 400 µL of precooled anhydrous methanol. The mixture was sonicated in an ice bath for 20 min and then incubated overnight at −20°C. Following centrifugation at 16 000 × g and 4°C for 20 min, the supernatant was dried using a high‐speed vacuum concentrator. The dried residue was reconstituted in 100 µL of 50% methanol in water and centrifuged at 20 000 × g and 4°C for 15 min. The final supernatant was collected for mass spectrometry analysis.

##### 4.2.4.2 UHPLC‐MS Analysis

The LC‐MS analysis was performed using a Shimadzu Nexera X2 LC‐30AD system coupled with an ACQUITY UPLC HSS T3 column (1.7 µm, 2.1 × 100 mm, Waters) and a 5500 QTRAP triple quadrupole mass spectrometer (AB SCIEX, Framingham, USA). Samples (2 µL) were injected via an autosampler and separated at 40°C with a flow rate of 200 µL min^−1^. The mobile phase consisted of 0.1% formic acid in water (solvent A) and acetonitrile (solvent B) with the following gradient: 100% A for 2.5 min, linear decrease to 70% A over 9 min, then to 0% A over 1 min, held for 5.4 min, followed by a 0.1 min return to initial conditions and 2.5 min re‐equilibration. QC samples were analyzed intermittently to monitor instrument stability.

Metabolites were detected in positive and negative electrospray ionization modes using the previously described multiple‐reaction monitoring (MRM) [53, 54]. Transitions from the MT1000 metabolite kit (Shanghai BioProfile, China) were optimized for declustering potential and collision energy. MS source parameters were: source temperature 550°C; Gas 1, 40 psi; Gas 2, 50 psi; curtain gas, 35 psi; ion spray voltage, ±4500 V (negative/positive mode, respectively).

#### 4.2.5 Targeted Metabolomics Analysis of Melibiose

Metabolite extraction and sample preparation for human plasma, mouse plasma, and bacterial culture supernatants were performed identically. Briefly, 100 µL aliquots were thawed at 4°C and mixed with 400 µL of precooled anhydrous methanol. The mixture was sonicated in an ice bath for 20 min and then incubated at 4°C for 2 h. Subsequently, the samples were centrifugated at 16 000 × g and 4°C for 20 min. The resulting supernatant was transferred and dried using a high‐speed vacuum concentrator. The dried residue was reconstituted with 50 µL of 50% (v/v) aqueous methanol, and then centrifuged at 20 000 × g and 4°C for 15 min. The final supernatant was collected for LC‐MS/MS analysis.

LC‐MS/MS analysis of melibiose was performed using a Shimadzu Nexera X2 LC‐30AD UHPLC system (Shimadzu, Kyoto, Japan) coupled with a QTRAP 6500+ mass spectrometer (AB SCIEX, Framingham, USA) equipped with an electrospray ionization (ESI) source operated in positive ion mode. Chromatographic separation was achieved on a reverse‐phase column using 0.1% formic acid in water as mobile phase A and 0.1% formic acid in acetonitrile as mobile phase B. Samples were maintained at 4°C in an autosampler, with a column temperature of 40°C. The flow rate was 300 µL min^−1^, and the injection volume was 5 µL. The gradient elution program was as follows: 0–2 min, 5% B; 2–6 min, linear increase to 99% B; 6–8 min, 99% B; 8‐8.1 min, linear decrease to 5% B; 8.1–10 min, 5% B for column re‐equilibration. The ESI source conditions were set as follows: source temperature 550°C, ion source gas 1 (GAS1) 55 psi, ion source gas 2 (GAS2) 60 psi, curtain gas (CUR) 35 psi, and ion spray voltage floating (ISVF) at 5500 V. Melibiose was detected using the MRM mode. The MRM transition for melibiose was m/z 360.1 → 85.1, with a decluttering potential (DP) of 60 V and a collision energy (CE) of 32 eV. All parameters were optimized to ensure the sensitivity and specificity of melibiose detection in both plasma and bacterial supernatants.

#### 4.2.6 Expression of β‐Fructosidase From *R. intestinalis* in *E. coli* MG1655

The gly32 (WP_006856626.1)‐overexpressing *E. coli* MG1655 strain was constructed using CRISPR‐Cas9‐mediated homologous recombination at the attλ site. A repair fragment containing gly32 flanked by attλ homology arms was amplified from the gly32‐pUC57 plasmid using primers attλ‐up‐F (5’‐AAGGATTCCACGCTGCAGCACC‐3’) and attλ‐down‐R (5’‐CACGCGCTGGATATAGAACGTA‐3’). The fragment, along with an attλ‐targeting sgRNA plasmid (target: 5’‐TCAAGTTAGTATAAAAAAGC‐3’), was electroporated into *E. coli* MG1655 strains carrying a Cas9‐expressing plasmid. Recombinants were selected on kanamycin/spectinomycin plates and verified by colony PCR using attλ‐up‐F/attλ‐down‐R (insertion: 2325 bp; wild‐type: 482 bp), and internal primers gly32‐cx‐F (5’‐TCTTCTGCCGTGAGAACAACTCC‐3’), gly32‐cx‐R (5’‐AACGAAGGTTTCTTCGCCGATCT‐3’), and gly32‐JD‐R (5’‐TGTTGATGTAAGCTGCGTGACGA‐3’). Helper plasmids were cured, and the resulting strain was designated MG1655‐gly32.

#### 4.2.7 Mouse Experiments

For the canagliflozin intervention study, a total of thirty‐two 8‐week‐old male mice, comprising *db/db* and *db/m* genotypes, were acclimated for one week. Eligible DKD mice (*db/db*, *n* = 24) were randomly divided into two groups according to whether canagliflozin was administered. The mice in the treatment group were further randomized to receive either a low dose (20 mg kg^−1^ day^−1^, *n* = 8) or a high dose (40 mg kg^−1^ day^−1^, *n* = 8) of canagliflozin via oral gavage. The *db/m* mice were designated the normal control group (NC, *n* = 8), while untreated DKD mice served as the disease model group (*n* = 8). Both the NC and model groups received an equal volume of PBS by oral gavage. Fasting blood glucose levels and body weight were measured biweekly. After 8 weeks of treatment, urine, fecal, plasma, and tissue samples were collected for further analyses.

For the gut microbiota depletion and transplantation experiments, DKD mice were treated with a broad‐spectrum antibiotic cocktail (ampicillin (1 g L^−1^), neomycin (1 g L^−1^), metronidazole (1 g L^−1^), and vancomycin (0.5 g L^−1^)) administered in the drinking water for 14 days to deplete the gut microbiota (the diet, water, and bedding were all sterilized). This method has been extensively proven to effectively deplete indigenous intestinal microbiota and readily facilitate the colonization of donor‐derived gut bacteria [55, 56]. DKD mice subjected to gut microbiota depletion were subsequently treated with canagliflozin for 8 weeks (*n* = 6). DKD mice treated with (*n* = 6) or without (*n* = 6) canagliflozin were used as controls. For fecal microbiota transplantation, the gut microbiome from canagliflozin‐treated or untreated DKD donor mice (*n* = 8 per group, 16 total) was transferred into two separate groups of recipient mice (*n* = 8 per group, 16 total). The recipients were DKD mice that had undergone gut microbiota depletion using antibiotics. Two weeks after depletion, 200 µL of fecal suspension from the donor was administered via oral gavage once daily for 14 consecutive days, followed by administration every other day. Fecal samples from the recipient mice were collected at weeks 0 and 2 of the FMT experiment. After 8 weeks of treatment with canagliflozin or FMT, urine, plasma, and tissue samples were collected for subsequent analyses.

For the *R. intestinalis* colonization study, a total of 18 DKD mice were used and randomly assigned to the three experimental groups (*n* = 6 per group). One group received a daily oral gavage of live *R. intestinalis* suspended in glycerol at a concentration of 1 × 10^9^ CFUs. Another group received an equal dose of heat‐killed *R. intestinalis*, which was prepared by autoclaving the bacterial suspension at 121°C and 225 kPa for 15 min. The third group served as the untreated model control and received an equivalent volume of glycerol vehicle. After 8 weeks of treatment, urine, fecal, plasma, and tissue samples were collected for further analyses.

In the melibiose treatment study, a total of 32 mice were used. DKD mice were orally administered melibiose via gavage at doses of 0.5 g kg^−1^ day^−1^ (low dose) or 1.0 g kg^−1^ day^−1^ (high dose) or an equal volume of PBS for 8 weeks (*n* = 8 per group). Age‐matched *db/m* mice that received PBS served as the normal control (NC) group (*n* = 8). Additionally, diabetic mice induced by the HFD combined with STZ were treated with melibiose at 1.0 g kg^−1^ day^−1^ for 8 weeks (*n* = 6 per group, 18 total). After the treatment period, urine, plasma, and tissue samples were collected for further analyses.

Adeno‐associated virus serotype 2/9 (AAV2/9) was employed as the delivery system to achieve in vivo knockdown and overexpression of GLO1. The recombinant vectors, AAV2/9‐TIE‐m‐GLO1‐3xFlag‐mCherry (for overexpression), AAV2/9‐TIE‐mir30‐m‐GLO1‐mCherry (for knockdown), and the control vector AAV2/9‐TIE‐mCherry, were obtained from Shanghai HanHeng Biotechnology Co., Ltd. (Shanghai, China). The titers of the viral stocks were 1.8 × 10^12^ vg mL^−1^ (overexpression vector), 1.3 × 10^12^ vg mL^−1^ (knockdown vector), and 1.5 × 10^12^ vg mL^−1^ (control vector), respectively. In the GLO1 overexpression experiment, 18 mice were used. Diabetic mice received a total dose of 1.8 × 10^11^ viral genomes (vg) in 100 µL of AAV2/9 vectors via intrarenal pelvis injection (6 mice per group), with 6 normal mice serving as the NC group. In the GLO1 knockdown experiment, 24 diabetic mice were used (6 mice per group). All diabetic mice underwent intrarenal pelvis injection of a total dose of 1.3 × 10^11^ vg in 100 µL AAV2/9 vectors, followed by daily administration of melibiose (1.0 g kg^−1^) or PBS for 8 weeks prior to subsequent analyses.

Diabetic mouse models were established in male C57BL/6J‐GLO1^fl/fl^, Tek‐cre^−^ (referred to as GLO1^fl/fl^) and C57BL/6J‐GLO1^fl/fl^, Tek‐cre^+^ (referred to as GLO1^−/−^) mice by combining a HFD with low‐dose STZ injections, as described previously. A total of 36 mice were used and randomly divided into six experimental groups (*n* = 6 per group). Age‐matched male GLO1^fl/fl^ and GLO1^−/−^ mice fed a standard chow diet (CD) served as the control (CON) groups (GLO1^fl/fl^‐CON and GLO1^−/−^‐CON). Successfully induced diabetic GLO1^fl/fl^ and GLO1^−/−^ mice were randomly assigned to receive either melibiose (1.0 g kg^−1^ day^−1^) or PBS via oral gavage, resulting in four diabetic intervention groups: GLO1^fl/fl^‐DM, GLO1^fl/fl^‐MEL, GLO1^−/−^‐DM, and GLO1^−/−^‐MEL. The mice in both CON groups were also administered an equal volume of PBS. After 8 weeks of treatment, urine, plasma, and tissue samples were collected for subsequent analyses.

In the stachyose treatment study, a total of 30 mice were used. DKD model mice were orally administered stachyose at doses of 0.5 g kg^−1^ (low dose, *n* = 6), 1.0 g kg^−1^ (middle dose, *n* = 6), or 2.0 g kg^−1^ (high dose, *n* = 6) once daily or an equal volume of PBS (*n* = 6) for 8 weeks. The *db/m* mice that received PBS served as the normal control group (NC, *n* = 6). After 8 weeks of treatment, urine, plasma, and tissue samples were collected for subsequent analyses.

#### 4.2.8 FMT Experiment Preparation

Fresh fecal pellets from donor mice were collected under sterile conditions within 1 h of defecation and homogenized in sterile saline (20 mg mL^−1^). The suspension was vortexed, filtered through a 200 µm mesh, and centrifuged at 1000 rpm for 5 min at 4°C. The supernatant was centrifuged again at 8000 rpm for 5 min. The resulting pellet was washed twice with sterile PBS and resuspended in an equal volume of sterile 40% glycerol to yield a final fecal suspension containing 20% glycerol.

#### 4.2.9 Biochemical Assays in Mice

Fasting blood glucose levels were measured using a Roche glucometer (Roche Diagnostics, Mannheim, Germany) after a 6‐h fast (07:00‐13:00), following National Institutes of Health (NIH) guidelines. The UACR was measured using a DIRUI CS‐400 analyzer (DIRUI Industrial Co., Ltd., China) as the primary outcome measure. Cr and BUN levels in mice were measured using commercial assay kits (Creatinine, Cat. S03036; Urea, Cat. S03076) from Rayto Life and Analytical Sciences Co., Ltd. (Shenzhen, China) on an automated biochemical analyzer (Rayto Life and Analytical Sciences Co., Ltd., Shenzhen, China). Plasma TNF‐α and IL‐6 concentrations were quantified using Servicebio ELISA kits (TNF‐α, Cat. GEM0004; IL‐6, Cat. GEM0001; Wuhan, China). Plasma lipopolysaccharide (LPS) levels in mice were measured using an ELISA kit (Cat. SEB526Ge, Cloud‐Clone Corp., Wuhan, China). Methylglyoxal levels in mice were measured using Cloud‐Clone ELISA kits (Wuhan, China).

#### 4.2.10 Histological Examination

Mouse kidney samples were resected and fixed in 4% paraformaldehyde for 24 h, followed by paraffin embedding and sectioning. The sections were stained with hematoxylin and eosin (H&E) for morphology, Masson for collagen deposition, and periodic acid‐Schiff (PAS) for glomerular basement membrane and extracellular matrix, followed by analysis with a Nexcope NE910 microscope (Ningbo Yongxin Optics Co., Ltd., Ningbo, China).

#### 4.2.11 Transmission and Scanning Electron Microscopy Examination

For transmission electron microscopy (TEM), fresh renal cortical tissues were fixed in 4°C precooled 2.5% glutaraldehyde for 48 h, dehydrated through a graded ethanol series, infiltrated with resin, and embedded. Ultrathin sections were mounted on copper grids, double‐stained with uranyl acetate and lead citrate, and examined under a transmission electron microscope for ultrastructural analysis. For scanning electron microscopy (SEM), fixed renal cortical tissues were dehydrated, dried, and sputter‐coated with gold. The samples were observed under a scanning electron microscope under high vacuum, and images were captured for morphological assessment.

#### 4.2.12 Immunofluorescence Staining

Paraffin‐embedded kidney sections were deparaffinized, rehydrated, and blocked with 5% bovine serum albumin (BSA) at room temperature for 30 min in the dark. Sections were then incubated with anti‐CD31 antibody (Cat. afrm0001, AiFang biological, Hunan, China) and anti‐ZO‐1 antibody (Cat. 21773‐1‐AP, Proteintech, Wuhan, China) overnight at 4°C. After washing, sections were incubated with fluorophore‐conjugated secondary antibodies (1:200 dilution) for 1 h at room temperature, followed by nuclear staining with DAPI. An anti‐fade mounting medium was applied to preserve fluorescence. Images were acquired using an inverted fluorescence microscope (Leica Microsystems, Wetzlar, Germany).

#### 4.2.13 Analysis of the Relative Abundances of *R. intestinalis* and β‐Fructosidase

Microbial genomic DNA was extracted from fecal samples of both human subjects and mice using the TIANamp Stool DNA Kit (Cat. DP328‐02; TIANGEN Biotech Co., Ltd., Beijing, China) according to the manufacturer's instructions. The DNA concentration and purity were assessed using a NanoDrop 2000 spectrophotometer (Thermo Fisher Scientific, Waltham, MA, USA). All the DNA samples were diluted to the same concentration and subjected to SYBR Green‐based quantitative real‐time PCR. Specific primers targeting *R. intestinalis* (forward: 5′‐GCATGACCTGGTGTGAA‐3’; reverse: 5’‐TTGGGCCGTGTCTCA‐3’) and β‐fructosidase (forward: 5’‐GACACTGTGGTTAGAGGAA‐3’; reverse: 5’‐CCTGTATTGCTGTAAGTCTG‐3’) were used. The relative abundances of the *R. intestinalis* and β‐fructosidase genes were normalized to the total bacterial 16S rRNA gene expression. The primer sequences are provided in Table S6.

#### 4.2.14 RNA Preparation and Real‐Time Quantitative PCR

Total RNA was extracted from homogenized kidney tissues using lysis buffer, followed by sequential phase separation with chloroform and precipitation with isopropanol. The RNA pellet was washed with 75% ethanol and dissolved in RNase‐free water. The RNA concentration and purity were assessed using a NanoDrop 2000 spectrophotometer (Thermo Fisher Scientific, Waltham, USA). cDNA was synthesized using a reverse transcription kit (Cat. AG11619, Accurate Biology, Hunan, China). According to the manufacturer's instructions, real‐time qPCR was performed using the SYBR Green Pro Taq HS Premixed qPCR Kit (Cat. AG11732, Accurate Biotechnology (Hunan) Co., Ltd., China). For the detection of gene expression, the relative mRNA expression level was calculated after normalization to β‐actin expression (in mice). The primers used are listed in Table S6.

#### 4.2.15 Transcriptome Sequencing (RNA‐Seq) Analysis

Total RNA was extracted from HGECs, and mRNA was enriched using oligo(dT) beads. After library preparation and PCR amplification, sequencing was performed on the Illumina NovaSeq platform. Read quality was assessed using FastQC, and the clean reads were aligned to the reference genome using HISAT2. A differential expression analysis was subsequently conducted. Gene set enrichment analysis (GSEA) was performed using the clusterProfiler package (V4.2.2) in R. Gene sets were obtained from the MSigDB database, and the enrichment results were visualized.

#### 4.2.16 GLO1 Overexpression, Knockdown, and Mutant Plasmid Transfection In Vitro

For GLO1 overexpression, HGECs were transfected with either the GLO1 overexpression plasmid or an empty pEX‐1 vector (GeneChem, Shanghai, China) using Lipofectamine 3000 reagent (Thermo Fisher Scientific, Waltham, USA) and incubated for 4 h.

For GLO1 knockdown, HGECs were infected with a lentivirus carrying GLO1‐targeting short hairpin RNA (shRNA; HanHeng Biotechnology, Shanghai, China) and cultured for the entire experimental period to ensure stable gene silencing. These GLO1‐knockdown cells were subsequently transfected with plasmids encoding wild‐type (WT) or mutant (R38A, N104A, E111A, K157A, M158A; HanHeng Biotechnology, Shanghai, China) GLO1 to identify the binding sites of melibiose on GLO1. The shRNA target sequence and details of the expression plasmids are listed in Table S7.

For GLO1 transient knockdown in MPC‐5 mouse podocytes and SV40 MES 13 mouse mesangial cells, cells were transfected with either the GLO1‐targeting siRNA or non‐targeting siRNA (HanHeng Biotechnology, Shanghai, China) using Lipofectamine 3000 reagent (Thermo Fisher Scientific, Waltham, USA) and incubated for 4 h. The siRNA sequences used are provided in Table S8.

#### 4.2.17 Drug Affinity Responsive Target Stability (DARTS) Assay

Human glomerular endothelial cell lysates (200 µL per sample) were aliquoted and incubated with either 2 µL DMSO (vehicle control) or 2 µL melibiose stock solution (50 µM final concentration) at room temperature for 1 h. Pronase was then added at a dilution of 1:300, followed by a 30‐min incubation at room temperature. Proteolysis was halted by adding a 20× protease inhibitor cocktail, and samples were chilled on ice, heat‐denatured, and prepared for SDS‐PAGE analysis.

Gel bands were destained, dehydrated, reduced with DTT, alkylated with iodoacetamide, and digested with trypsin overnight at 37°C. The resulting peptides were extracted and desalted using C18 ZipTips and reconstituted in 0.1% formic acid. Peptide samples were then analyzed by liquid chromatography‐tandem mass spectrometry (LC‐MS/MS) using an EASY‐nLC 1200 system coupled with a Q‐Exactive HF mass spectrometer (Thermo Fisher Scientific, Waltham, USA).

#### 4.2.18 Cellular Thermal Shift Assay (CETSA)

Human glomerular endothelial cells were lysed in PBS supplemented with a protease inhibitor cocktail. The cell suspension was flash‐frozen in liquid nitrogen and subjected to three freeze‐thaw cycles. Then, the mixture was centrifuged at 20 000 g for 15 min at 4°C to collect the supernatants, which were then split into two equal parts and incubated with or without 50 µM melibiose for 30 min at room temperature. Subsequently, the samples were incubated at varying temperatures (37°C, 40°C, 43°C, 46°C, 49°C, 52°C, 55°C, 58°C) for 3 min each, then protein stability was determined by western blotting.

#### 4.2.19 Microscale Thermophoresis (MST) Assay

MST analysis was performed using the Monolith NT.115 system (NanoTemper Technologies, Munich, Germany). Purified GLO1 protein (Cat. ab206792, Abcam, Cambridge, UK) was fluorescently labeled with the RED‐NHS Protein Labeling Kit (Cat. MO‐L011, NanoTemper Technologies, Munich, Germany) according to the manufacturer's instructions. A series of 16‐point serial dilutions of melibiose (Cat. T2914, Taoshu Biotechnology, Shanghai, China) were prepared in 50 mM HEPES buffer (pH 7.4) containing 0.05% Tween‐20 and incubated with an equal volume of labeled protein at room temperature for 20 min. The mixtures were then loaded into MST Premium Capillaries, and thermophoresis was measured using standard parameters. The dissociation constant (Kd) was calculated using NanoTemper analysis software.

#### 4.2.20 Molecular Docking

The chemical structure of melibiose was obtained from the PubChem database and subjected to energy minimization using Chem3D software. The crystal structure of human GLO1 (PDB ID: 3W0T; 1.35 Å resolution) [57] was downloaded from the UniProt database. Water molecules and ligands were removed using PyMOL, and the protein structure was converted using AutoDockTools 1.5.6. Following ligand and receptor preparation, molecular docking was performed using AutoDock Vina to predict binding affinity and potential interaction sites.

#### 4.2.21 GLO1 Enzyme Activity Detection

GLO1 enzymatic activity was measured using a commercial assay kit (JL‐T1284, JonlnBio, Shanghai, China) according to the manufacturer's instructions. Briefly, cell pellets were lysed in extraction buffer on ice, followed by centrifugation at 12 000 rpm for 10 min at 4°C. The supernatant was collected, mixed with the reaction solution, and incubated at room temperature. Absorbance at 240 nm was recorded at 30 s (A_1_) and 5 min (A_2_) using a UV spectrophotometer. The change in absorbance (ΔA = A_2_ − A_1_) reflects the formation of S‐D‐lactoylglutathione. One unit of GLO1 activity was defined as the amount of enzyme that catalyzes the formation of 1 nmol of S‐D‐lactoylglutathione per minute per milligram of protein under assay conditions.

#### 4.2.22 Western Blotting

Cells and renal tissue homogenates were lysed in RIPA buffer containing protease and phosphatase inhibitors. The total protein contents were quantified using a BCA assay kit (Cat. DQ111‐01, TransGen Biotech, Beijing, China). Protein samples were denatured in SDS buffer, separated by 8% SDS‐PAGE, and then transferred onto polyvinylidene fluoride (PVDF) membranes (Millipore, Billerica, MA). After blocking with protein‐free blocking buffer (Cat. P0240, Beyotime, Shanghai, China) for 5–10 min at room temperature, and blots were incubated with various primary antibodies overnight at 4°C, including ZO‐1 (1:5000, 21773‐1‐AP, RRID: AB_10733242, Proteintech), OCLN (1:5000, 27260‐1‐AP, AB_2880820, Proteintech), SDC1 (1:2000, ab128936, RRID: AB_11150990, Abcam), ET‐1 (Mouse, 1:1000, GB11557‐100, Servicebio; Human, 1:1000, 12191‐1‐AP, RRID: AB_889392, Proteintech), ICAM‐1 (Mouse, 1:1000, GB11106‐100, Servicebio; Human, 1:10000, 10831‐1‐AP, RRID: AB_2264494, Proteintech), and GLO1 (1:5000,15140‐1‐AP, RRID: AB_2109890, Proteintech), followed by a horseradish peroxidase‐conjugated secondary antibody for another 1 h. Protein bands were detected using enhanced chemiluminescence (ECL, Cat. P10300, New Cell & Molecular Biotech, Suzhou, China) and imaged with a Chemiluminescence imaging system (SCG‐W2000, Servicebio, Wuhan, China). Band intensities were quantified using ImageJ software.

### 4.3 Quantification and Statistical Analysis

#### 4.3.1 Statistical Analysis

For clinical data, analyses were performed with IBM SPSS Statistics version 26.0 (IBM Corp.) as two‐sided test with a significance level of α = 0.05. The normality of the data was assessed using the Kolmogorov–Smirnov test. Normally distributed continuous variables are presented as the means ± standard deviations (SDs), whereas non‐normally distributed variables are presented as medians and interquartile ranges. For Cohort 1, Within‐group comparisons (CON or CANA) were performed using paired two‐tailed Student's *t* tests for normally distributed variables or Wilcoxon signed‐rank tests for non‐normally distributed variables. Between‐group comparisons were conducted using unpaired Student's *t* tests for parametric data or Mann–Whitney U tests for nonparametric data. Categorical variables were analyzed using the Pearson's chi‐square test; Fisher's exact test was employed when expected cell counts were less than 5. For Cohort 2, a paired two‐tailed Student's *t* test or Wilcoxon signed‐rank test was used, depending on the data distribution.

For the animal, cell line, and bacterial experiments, all analyses were performed with GraphPad Prism version 9.5 (GraphPad Software). Group sizes commonly used in murine studies were applied. For physiological measurements and mouse tissue analyses, the sample size refers to the number of individual mice included in each analysis, as specified in the corresponding figure legends. Comparisons between two groups were performed using an unpaired two‐tailed Student's *t*‐test. Comparisons among more than two groups were performed using one‐way ANOVA followed by Tukey's post hoc test. For experiments involving two independent variables, two‐way ANOVA followed by Tukey's post hoc multiple comparisons test was used. Unless otherwise indicated, all tests were two‐tailed, and *p* < 0.05 was considered statistically significant.

#### 4.3.2 Microbiome Data

The bioinformatics analysis of the microbiome was performed using QIIME2 (version 2019.4) with modifications based on official tutorials (https://docs.qiime2.org/2019.4/tutorials/). Alpha diversity indices (Chao1 and Simpson indices) in each sample at the genus level were determined with the R package *vegan*. Principal coordinate analysis (PCoA) based on the Bray‐Curtis distance and PERMANOVA based on the Adonis function distance were performed using the R package *vegan* V2.6.4 to deduce the differences in community diversity between samples. The taxonomic composition was profiled based on the ASV classification. Microbial abundance at the phylum and genus levels was visualized using the R package (V4.2.1). Differentially abundant taxa were identified using the linear discriminant analysis effect size (LEfSe) method implemented in the microeco package. A Pearson correlation analysis of the relative abundance of key taxa and clinical indicators was conducted.

Species‐level relative abundances of *Roseburia* were extracted from the microbial composition data using the microeco package in R (v4.2.1). To control for the potential confounding effects of concomitant antihyperglycemic therapy on the gut microbiota, an analysis of covariance was performed, adjusting for the use of these drugs (as binary covariates). Linear regression model was explored to examine the associations between *Roseburia* species abundance and UACR. The correlation coefficient (R) and corresponding *p*‐value were calculated to assess statistical significance.

#### 4.3.3 Metabolomic Data

The raw MRM data of the MT1000 KIT metabolites were processed using MultiQuant software V3.0.2 to extract peak areas for quantification across different samples. Discriminative metabolites between groups were identified based on variable importance in projection (VIP) values from the orthogonal partial least squares discriminant analysis (OPLS‐DA) model and *p* values from two‐tailed Student's *t* tests of the peak areas. Metabolites with VIP > 1 and *p* < 0.05 were considered statistically significant. The top 10 differentially abundant metabolites ranked by *p* value were selected, and their within‐group changes (pre‐ vs. post‐treatment) were visualized as log_2_fold changes (log_2_FCs) in the treatment and control groups. Pearson's correlation analysis was performed to assess the associations between these metabolites and clinical parameters. A linear regression model was applied to evaluate the relationship between melibiose and *Roseburia intestinalis* abundance.

## Author Contributions

W.Z., Y.S., C.‐K.L., Y.‐Y.L., M.‐W.S., F.G., Y.‐Y.Z. and G.‐J.Q. conceived the study and interpreted the data. W.Z., Y.S., C.‐K.L., Y.‐Y.L., F.G., F.‐Y.W., X.‐J.F., and W.‐W.G. drafted and revised the manuscript. G.‐J.Q., Y.‐Y.Z., and Y.S. contributed to funding acquisition. W.Z., Y.‐Y.L., and C.‐K.L. performed and interpreted 16S rRNA sequencing, the metabolomic analysis, transcriptome analysis, and mass spectrometry analysis. W.Z., Y.‐Y.L., M.‐W.S., F.G., F.‐M.X., Y.‐H.S., D.‐M.Z., Y.‐H.Z., L.‐W.W., Z.‐Q.K., Y.‐J.Y., Y.‐X.L., X.‐J.M., J.W., C.L., S.‐N.M., L.Z., Z.Q., G.‐L.X., Q.‐B.Z., J. L., S.‐M.S., D.Z., T.H., and Q.‐Z.W. contributed to participant recruitment, sample collection, and biobank management. W.Z., Y.‐Y.L., Y.S., F.‐Y.W., X.‐J.F., and W.‐W.G. participated in the construction of diabetic kidney disease and diabetic animal models, Abx treatment, *R. intestinalis* colonization, FMT, melibiose supplementation, and transgenic mouse experiments. W.Z. and Y.S. conducted and interpreted in vitro cell experiments, ROS detection, GLO1 activity assays, and genetic manipulations both in vivo and in vitro. C.‐K.L. performed bacterial culture, genetic modification of bacterial strains, and HPLC‐MS/MS analysis. W.Z., Y.S., Y.‐Y.L., and C.‐H.S. conducted statistical analyses. G.‐J.Q., Y.‐Y.Z., Y.S., M.‐W.S., F.G., C.‐H.S., and G.‐L.X. provided critical comments on the manuscript. All authors discussed and approved the manuscript.

## Funding

This work was supported by the following grants: National Natural Science Foundation of China (82170839 and U23A20414, G.Q.; 82470876, Y.Z.; 82300930, Y.S.), Key Research and Development Program of Henan Province (251111311500, G.Q.), Joint Construction Project of Henan Medical Science and Technology Research Plan Project (SBGJ202301006, G.Q.), Natural Science Foundation of Henan Province (242300421272, Y.Z.), and Henan Joint Construction Program (LHGJ20230200, Y.S.).

## Conflicts of Interest

The authors declare no conflicts of interest.

## Supporting information




**Supporting File**: advs75842‐sup‐0001‐SuppMat.docx.

## Data Availability

The data that support the findings of this study are available from the corresponding author upon reasonable request.
