# Supplementary material for: Canagliflozin Alleviates Diabetic Glomerular Endothelial Injury via Melibiose in a Microbiota‐Dependent Manner
Source: Adv Sci (Weinh). 2026 May 27;13(47):e17222. doi: 10.1002/advs.202517222 (PMC13335814; doi:10.1002/advs.202517222)
Supplement: Supplementary file 1 [file ADVS-13-e17222-s001.docx]

Supporting Information

**Canagliflozin Alleviates Diabetic Glomerular Endothelial Injury via Melibiose in a Microbiota-dependent Manner**

*Wei Zhang*^1,2,16^*, Yi Song*^1,2,16^*, Changkun Li*^3,16^*, Yuanyuan Luo*^1,2^*, Mingwei Shao*^1^*, Feng Guo*^1,2^*, Fangyi Wei*^1,2^*, Xunjie Fan*^1,2^*, Wenwen Guo*^1,2^*, Fengmei Xu*^4^*, Yanhong Sang*^5^*, Dongming Zhang*^6^*, Yanhong Zhou*^7^*, Lianwei Wang*^8^*, Zhiqiang Kang*^9^*, Yingjun Yang*^10^*, Chunhua Song*^11^*, Yanxia Liu*^1^*, Xiaojun Ma*^1^*, Jiao Wang*^1^*, Chong Li*^1^*, Shengnan Ma*^1^*, Lin Zhao*^1^*, Zhi Qin*^12^*, Guolan Xing*^13^*, Qiubo Zhao*^4^*, Jun Li*^6^*, Shumin Song*^5^*, Dan Zhao*^8^*, Ting Huang*^10^*, Qingzhu Wang*^14^*, Yanyan Zhao*^1,15^** & Guijun Qin*^1,2^***

**Author affiliations**

1. Department of Endocrinology and Metabolism, The First Affiliated Hospital of Zhengzhou University, Zhengzhou, 450052, China.
2. Tianjian Laboratory of Advanced Biomedical Sciences, Zhengzhou, 450001, China.
3. Department of Endocrine and Metabolic Diseases, Shanghai Institute of Endocrine and Metabolic Diseases, Ruijin Hospital, Shanghai Jiao Tong University School of Medicine, Shanghai, 200025, China.
4. Department of Endocrinology and Metabolism, Hebi Coal Industry (Group) Co., Ltd. General Hospital, Hebi, 458000, China.
5. Department of Endocrinology and Metabolism, The Fifth Affiliated Hospital of Zhengzhou University, Zhengzhou, 450052, China.
6. Department of Endocrinology and Metabolism, The Second Affiliated Hospital of Zhengzhou University, Zhengzhou, 450003, China
7. Department of Endocrinology, Xinxiang Central Hospital, Xinxiang, 453002, China.
8. Department of Endocrinology, Zhumadian Central Hospital, Zhumadian, 463000, China.
9. Department of Endocrinology and Metabolism, Zhengzhou Central Hospital, Zhengzhou, 450007, China.
10. Department of Endocrinology and Geriatrics, The Seventh People's Hospital of Zhengzhou, Zhengzhou, 450047, China.
11. Department of Epidemiology and Statistics, College of Public Health, Zhengzhou University, Zhengzhou, 450001, China.
12. Department of Thoracic Surgery, The First Affiliated Hospital of Zhengzhou University, Zhengzhou, 450052, China.
13. Department of Nephrology, The First Affiliated Hospital of Zhengzhou University, Zhengzhou, 450052, China.
14. Department of Nuclear Medicine, The First Affiliated Hospital of Zhengzhou University, Zhengzhou, 450052, China.
15. Henan Provincial Center for Diabetes Prevention and Control, Zhengzhou, 450052, China.
16. These authors contributed equally: Wei Zhang, Yi Song, Changkun Li.

*Corresponding authors:

Guijun Qin, PhD

Department of Endocrinology and Metabolism

The First Affiliated Hospital of Zhengzhou University

450052 Zhengzhou, China

Email: hyqingj@zzu.edu.cn

Yanyan Zhao, PhD

Department of Endocrinology and Metabolism

The First Affiliated Hospital of Zhengzhou University

450052 Zhengzhou, China

Email: [fcczhaoyy1@zzu.edu.cn](mailto:fcczhaoyy1@zzu.edu.cn)

**Supplementary Figure S1 CONSORT diagram.**

CONSORT flow diagram illustrating the procedures for screening, intervention allocation, follow-up, and data analysis of the study. CON, control DKD patients; CANA, canagliflozin-treated DKD patients.

**Supplementary Figure S2 Canagliflozin alters gut microbiota composition at phylum and genus levels.**

A,B) Comparison of alpha-diversity indices (Chao1 index and Simpson index) among the CON group and CANA group.

C,D) Stacked bar plots showing the relative abundance of phylum (C) and genus (D) in the CON group and the CANA group.

E,F) Fasting blood glucose (E) and body weight (F) levels in mice (*n* = 8).

G,H) Creatinine (Cr) (G) and blood urea nitrogen (BUN) (H) levels in mice (*n* = 8).

I-M) Quantitative analysis of ET-1 (I), SDC1 (J), OCLN (K), ICAM-1 (L) and ZO-1 (M) expression levels by Western blot (*n* = 4).

N,O) Stacked bar plots showing the relative abundance of phylum (N) and genus (O) in the NC, DKD, and CANA groups.

Data are presented as mean ± SD (A, B, E-M). Statistical analysis was performed using two-way ANOVA (E, F) and one-way ANOVA (G-M). **p* < 0.05, ***p* < 0.01, ****p* < 0.001, *****p* < 0.0001, ns *p* > 0.05.

**Supplementary Figure S3 Fasting blood glucose and body weight in canagliflozin-treated pseudo-germ-free and FMT recipient mice.**

A) Representative images of fecal supernatants from DKD mice receiving Abx or normal drinking water, plated on LB agar and incubated overnight at 37 °C.

B,C) Fasting blood glucose (B) and body weight (C) levels in mice (*n* = 6).

D-H) Quantitative analysis of ET-1 (D), SDC1 (E), OCLN (F), ICAM-1 (G) and ZO-1 (H) expression levels by Western blot (*n* = 4).

I) Representative images of fecal supernatants from DKD mice given normal water and recipient DKD mice given Abx water, plated on LB agar and incubated overnight at 37 °C.

J,K) Fasting blood glucose (J) and body weight (K) levels in mice (*n* = 8).

L-P) Quantitative analysis of ET-1 (L), SDC1 (M), OCLN (N), ICAM-1 (O) and ZO-1 (P) expression levels by Western blot (*n* = 3).

Data are presented as mean ± SD (B-H, J-P). Statistical analysis was performed using the two-way ANOVA (B, C, J, K), one-way ANOVA (D-H) and the unpaired two-tailed Student’s test (L-P). **p* < 0.05, ***p* < 0.01, ****p* < 0.001, *****p* < 0.0001.

**Supplementary Figure S4 Melibiose ameliorates glomerular endothelial injury in diabetic mice.**

A-E) Quantitative analysis of ET-1 (A), SDC1 (B), OCLN (C), ICAM-1 (D) and ZO-1 (E) expression levels by Western blot (*n* = 4).

F) Experimental scheme for melibiose intervention. Diabetic mice induced by a high-fat diet combined with STZ received melibiose (1.0 g kg^-1^) or PBS for 8 weeks, and age-matched mice fed a chow diet were administered PBS as controls.

G,H) Fasting blood glucose (G) and body weight (H) levels in mice (*n* = 6).

I-K) UACR (I), Cr (J), and BUN (K) levels in mice (*n* = 6).

L) Representative images of H&E, Masson's trichrome, and PAS staining showing DKD-associated glomerular pathology. Scale bar, 20 μm.

M) Representative TEM and SEM images showing DKD-associated glomerular endothelial injury. Scale bar, 500 nm.

N) Morphometric quantification of the endothelial cell fenestration density per unit length, performed on TEM images (*n* = 3).

O) Morphometric quantification of the endothelial cell fenestration area density, performed on SEM images (*n* = 3).

P) Representative immunofluorescence images of glomeruli showing CD31 (red) staining for endothelial cells and DAPI (blue) staining for nuclei in DKD mice. Scale bar, 20 μm.

Q) Quantitative analysis of CD31 fluorescence intensity in glomeruli of DKD mice (*n* = 3).

R-W) Representative immunoblotting (R) and quantitative analysis of ET-1 (S), SDC1 (T), OCLN (U), ICAM-1 (V) and ZO-1 (W) proteins in the kidney (*n* = 3).

Data are presented as mean ± SD (A-E, G-K, N, O, Q, S-W). Statistical analysis was performed using the two-way ANOVA (G, H) and the one-way ANOVA (A-E, I-K, Q, S-W). **p* < 0.05, ***p* < 0.01, ****p* < 0.001, *****p* < 0.0001.

**Supplementary Figure S5 Melibiose ameliorates glomerular endothelial injury in glomerular endothelial cells under high-glucose conditions.**

A) CCK-8 assay of HGECs under high-glucose conditions with melibiose treatment (0-300 μ_M_) (*n* = 5).

B-F) Representative immunoblotting (B) and quantitative analysis of ET-1 (C), OCLN (D), ICAM-1 (E) and ZO-1 (F) proteins in HGECs (*n* = 3).

G,H) Representative DCFH-DA fluorescence images showing intracellular ROS levels in HGECs (G) and the corresponding quantitative analysis of fluorescence intensity (H).

Data are presented as mean ± SD (A, C-F, H). Statistical analysis was performed using the one-way ANOVA (A, C-F, H). **p* < 0.05, ***p* < 0.01, ****p* < 0.001, *****p* < 0.0001.

**Supplementary Figure S6 Melibiose suppresses AGE-RAGE downstream effectors (TNF-α and IL-6) and identifies GLO1 as a direct binding target.**

A-D) Plasma TNF-α (A,C) and plasma IL-6 (B,D) levels in mice (*n* = 6).

E-H) Renal TNF-α (E,G) and IL-6 (F,H) mRNA expression in diabetic mice (*n* = 3).

I,J) ELISA analysis of TNF-α (I) and IL-6 (J) concentrations in the supernatants of HGECs (*n* = 3).

K) SDS-PAGE gel showing Pronase-dependent proteolysis of HGEC lysates for DARTS assay optimization.

L) SDS-PAGE gel showing Pronase digestion of HGEC lysates with or without melibiose treatment (*n* = 3).

M) Representative immunoblot and quantification of GLO1 protein levels in HGECs under NG, HG or melibiose-treated (25 μ_M_ and 50 μ_M_) conditions (*n* = 3).

N) Representative immunoblot and quantification of GLO1 protein levels in HGECs transfected with three different GLO1-targeting shRNAs (shRNA#1, shRNA#2, and shRNA#3) to assess knockdown efficiency (*n* = 3).

Data are presented as mean ± SD (A-J, M, N). Statistical analysis was performed using the one-way ANOVA (A-J, M, N). **p* < 0.05, ***p* < 0.01, ****p* < 0.001, *****p* < 0.0001, ns *p* > 0.05.

**Supplementary Figure S7 Melibiose ameliorates podocyte and mesangial cell injury through GLO1 under high-glucose conditions.**

A,B) Representative immunoblots (A) and quantitative analysis (B) of GLO1 in HGECs, MPC-5, and SV40 MES 13 (*n* = 3).

C-F) Representative immunoblots (C) and quantitative analysis of GLO1 (D), Podocin (E) and Nephrin (F) in MPC-5 (*n* = 3).

G-J) Representative immunoblots (G) and quantitative analysis of GLO1 (H), α-SMA (I) and Collagen I (J) in SV40 MES 13 (*n* = 3).

Data are presented as mean ± SD (B, D-F, H-J). Statistical analysis was performed using the one-way ANOVA (B, D-F, H-J). **p* < 0.05, ***p* < 0.01, ****p* < 0.001, ns *p* > 0.05.

**Supplementary Figure S8 Endothelial-specific GLO1 overexpression alleviates glomerular endothelial injury in diabetic mice.**

A) Experimental scheme of diabetic mouse model with GLO1 overexpression. Diabetes was induced by high-fat diet and STZ injection, followed by kidney-targeted overexpression of GLO1 via AAV delivery under the control of the endothelial-specific Tie2 promoter.

B,C) Fasting blood glucose (B) and body weight (C) levels in mice (*n* = 6).

D-F) UACR (D), Cr (E), and BUN (F) levels in mice (*n* = 6).

G,H) Plasma TNF-α (G) and plasma IL-6 (H) levels in mice (*n* = 6).

I) Methylglyoxal levels in mouse kidney (*n* = 4-5).

J) Representative images of H&E, Masson's trichrome, and PAS staining showing DKD-associated glomerular pathology. Scale bar, 20 μm.

K) Representative TEM and SEM images showing glomerular endothelial fenestration structures. Scale bar, 500 nm.

L) Morphometric quantification of the endothelial cell fenestration density per unit length, performed on TEM images (*n* = 3).

M) Morphometric quantification of the endothelial cell fenestration area density, performed on SEM images (*n* = 3).

N-S) Representative immunoblotting (N) and quantitative analysis of ET-1 (O), SDC1 (P), OCLN (Q), ICAM-1 (R) and ZO-1 (S) proteins in the kidney (*n* = 3).

Data are presented as mean ± SD (B-I, L, M, O-S). Statistical analysis was performed using the two-way ANOVA (B, C) and the one-way ANOVA (D-I, L, M, O-S). **p* < 0.05, ***p* < 0.01, ****p* < 0.001, *****p* < 0.0001, ns *p* > 0.05.

**Supplementary Figure S9 Endothelial-specific GLO1 knockdown attenuates the protective effect of melibiose on glomerular endothelial injury in diabetic mice.**

A) Experimental scheme of diabetic mouse model with GLO1 knockdown. Diabetes was induced by high-fat diet and STZ injection, followed by kidney-targeted knockdown of GLO1 via AAV delivery under the control of the endothelial-specific Tie2 promoter.

B,C) Fasting blood glucose (B) and body weight (C) levels in mice (*n* = 6).

D-F) UACR (D), Cr (E), and BUN (F) levels in mice (*n* = 6).

G,H) Plasma TNF-α (G) and plasma IL-6 (H) levels in mice (*n* = 6).

I) Methylglyoxal levels in mouse kidney (*n* = 4-5).

J) Representative images of H&E, Masson's trichrome, and PAS staining showing DKD-associated glomerular pathology. Scale bar, 20 μm.

K) Representative TEM and SEM images showing glomerular endothelial fenestration structures. Scale bar, 500 nm.

L) Morphometric quantification of the endothelial cell fenestration density per unit length, performed on TEM images (*n* = 3).

M) Morphometric quantification of the endothelial cell fenestration area density, performed on SEM images (*n* = 3).

N-S) Representative immunoblotting (N) and quantitative analysis of ET-1 (O), SDC1 (P), OCLN (Q), ICAM-1 (R) and ZO-1 (S) proteins in the kidney (*n* = 4).

Data are presented as mean ± SD (B-I, L, M, O-S). Statistical analysis was performed using the two-way ANOVA (B, C) and the one-way ANOVA (D-I, L, M, O-S). **p* < 0.05, ***p* < 0.01, ****p* < 0.001, *****p* < 0.0001, ns *p* > 0.05.

**Supplementary Figure S10 CONSORT diagram.**

CONSORT flow diagram illustrating the procedures for enrollment, intervention allocation, follow-up, and data analysis of the study.

**Supplementary Figure S11 Melibiose precursor elevates plasma melibiose and attenuates albuminuria in DKD mice.**

A) Experimental scheme for stachyose intervention. Male DKD mice received stachyose at 0.5 g kg^-1^, 1.0 g kg^-1^, 2.0 g kg^-1^, or PBS for 8 weeks, and age-matched non-DKD control mice were administered PBS.

B,C) Fasting blood glucose (B) and body weight (C) levels in mice (*n* = 6).

D) Plasma melibiose levels in mice (*n* = 6).

E-G) UACR (E), Cr (F), and BUN (G) levels in mice (*n* = 6).

H,I) Plasma TNF-α (H) and plasma IL-6 (I) levels in mice (*n* = 6).

J,K) Methylglyoxal levels in mouse kidney (J) and plasma (K) (*n* = 5-6).

Data are presented as mean ± SD (B-K). Statistical analysis was performed using the two-way ANOVA (B, C) and the one-way ANOVA (D-K). **p* < 0.05, ***p* < 0.01, ****p* < 0.001, *****p* < 0.0001, ns *p* > 0.05.

**Table S1 Summary of clinical parameters of the study participants before and after treatment**

| Characteristic | Control group (*n* = 85) | |  | Canagliflozin group (*n* = 85) | |  | *P* value^a^ | *P* value^b^ | *P* value^c^ | *P* value^d^ |
| --- | --- | --- | --- | --- | --- | --- | --- | --- | --- | --- |
|  | 0w | 26w |  | 0w | 26w |  |  |  |  |  |
| Sex [male:female] | 55:30 | - |  | 56:29 | - |  | 0.872 | - | - | - |
| Age [years] | 58.00 (52.00, 65.50) | - |  | 59.00 (50.00, 66.50) | - |  | 0.880 | - | - | - |
| Duration [years] | 10.00 (6.00, 15.00) | - |  | 10.00 (6.00, 18.00) | - |  | 0.709 | - | - | - |
| SBP [mmHg] | 130.00 (123.50, 140.00) | 128.00 (119.00, 137.50) |  | 133.00 (122.50,148.00) | 129.00 (120.00, 140.00) |  | 0.474 | 0.001 | ＜0.001 | 0.615 |
| DBP [mmHg] | 80.00 (74.50, 85.50) | 79.00 (72.00, 83.50) |  | 79.00 (73.50, 89.00) | 78.00 (73.50, 82.00) |  | 0.828 | 0.420 | 0.072 | 0.989 |
| BMI [kg m^-2^] | 25.35 (23.36, 27.70) | 25.10 (23.49, 27.23) |  | 25.71 (23.55, 27.89) | 25.56 (23.66, 27.45) |  | 0.360 | 0.057 | ＜0.001 | 0.995 |
| HbA_1c_ [%] | 8.40 (7.40, 9.40) | 7.10 (6.70, 7.70) |  | 8.30 (7.10, 9.30) | 7.10 (6.40, 7.70) |  | 0.435 | ＜0.001 | ＜0.001 | 0.685 |
| UACR [mg g^-1^] | 722.10 (537.54, 927.68) | 694.80 (474.15, 970.46) |  | 698.20 (558.65, 926.30) | 481.01 (334.35, 816.99) |  | 0.815 | 0.455 | ＜0.001 | ＜0.001 |
| Urea [mmol L^-1^] | 7.80 (6.70, 8.86) | 7.31 (6.70, 8.97) |  | 7.26 (6.44, 8.81) | 7.36 (6.40, 8.28) |  | 0.330 | 0.396 | 0.032 | 0.408 |
| Creatinine [μmol L^-1^] | 90.00 (81.00, 102.50) | 91.00 (81.00, 108.00) |  | 90.00 (78.00, 99.00) | 90.00 (79.50, 101.00) |  | 0.468 | 0.003 | 0.253 | 0.313 |
| Uric acid [μmol L^-1^] | 368.42 ± 10.77 | 340.77 ± 8.22 |  | 345.50 ± 11.07 | 315.36 ± 8.57 |  | 0.140 | 0.008 | 0.001 | 0.034 |
| eGFR [mL min^-1^ 1.73m^-2^] | 77.23 (63.26, 86.35) | 73.75 (59.28, 84.25) |  | 79.48 (66.02, 85.35) | 78.15 (64.55, 85.12) |  | 0.376 | 0.017 | 0.434 | 0.283 |
| TC [mmol L^-1^] | 4.53 (3.80, 5.72) | 4.23 (3.52, 4.78) |  | 4.56 (3.93, 5.83) | 4.03 (3.37, 4.76) |  | 0.629 | ＜0.001 | ＜0.001 | 0.210 |
| TG [mmol L^-1^] | 1.78 (1.22, 3.28) | 1.77 (1.09, 2.37) |  | 1.90 (1.31, 3.27) | 1.49 (1.09, 2.11) |  | 0.368 | 0.090 | ＜0.001 | 0.143 |
| HDL-C [mmol L^-1^] | 1.03 (0.90, 1.23) | 1.17 (0.95, 1.36) |  | 1.07 (0.91, 1.20) | 1.12 (1.00, 1.36) |  | 0.699 | 0.009 | 0.031 | 0.801 |
| LDL-C [mmol L^-1^] | 2.66 (2.03, 3.45) | 2.55 (1.99, 3.00) |  | 2.68 (1.94, 3.31) | 2.35 (1.88, 2.75) |  | 0.928 | 0.116 | ＜0.001 | 0.108 |

SBP, systolic blood pressure; DBP, diastolic blood pressure; BMI, body mass index; HbA1c, hemoglobin A1c; UACR, urine albumin-to-creatinine ratio; eGFR, estimated glomerular filtration rate; TC, total cholesterol; TG, triacylglycerol; HDL-C, high-density lipoprotein cholesterol; LDL-C, low-density lipoprotein cholesterol. Dates are expressed as mean ± s.d. or median (interquartile range, 25th-75th percentile). ^a)^Between-group differences at baseline between the CON and CANA groups (unpaired Student’s t test or Mann-Whitney test). ^b)^Within-group differences before and after intervention in the CON group (paired Student’s t test or Wilcoxon signed-rank test). ^c)^Within-group differences before and after intervention in the CANA group (paired Student’s t test or Wilcoxon signed-rank test). ^d)^Between-group differences after the intervention between the CON and CANA groups (unpaired Student’s t test or Mann-Whitney test).

**Table S2 Concomitant antihyperglycemic therapy at baseline and end of follow-up in Canagliflozin and Control groups (On-Treatment Set)**

|  | Baseline | | |  | End of follow-up | | |
| --- | --- | --- | --- | --- | --- | --- | --- |
| Participants, *n* [%] | Control group  (*n* = 85) | Canagliflozin group  (*n* = 85) | *P* value |  | Control group  (*n* = 85) | Canagliflozin group  (*n* = 85) | *P* value |
| Biguanides | 44 (51.8) | 53 (62.4) | 0.163 |  | 49 (57.6) | 53 (62.4) | 0.531 |
| DPP-4 inhibitors | 13 (15.3) | 16 (18.8) | 0.541 |  | 24 (28.2) | 16 (18.8) | 0.148 |
| Insulin | 29 (34.1) | 31 (36.5) | 0.748 |  | 39 (45.9) | 31 (36.5) | 0.213 |
| Alpha glucosidase inhibitors | 22 (25.9) | 21 (24.7) | 0.860 |  | 27 (31.8) | 21 (24.7) | 0.307 |
| Sulfonylureas | 7 (8.2) | 13 (15.3) | 0.153 |  | 14 (16.5) | 13 (15.3) | 0.834 |
| Thiazolidinediones | 2 (2.4) | 4 (4.7) | 0.682 ^a^ |  | 4 (4.7) | 4 (4.7) | 1.000 ^a^ |
| Glinides | 1 (1.2) | 2 (2.4) | 1.000 ^a^ |  | 2 (2.4) | 2 (2.4) | 1.000 ^a^ |

Data are presented as n [%]. Between-group comparisons were made using Pearson's chi-square test; ^a)^Fisher’s exact test (two-sided) was used due to expected cell counts less than 5.

**Table S3 Adjusted and unadjusted analysis of *Roseburia* abundances between Canagliflozin and Control groups at end of follow-up**

|  | *P* value (Unadjusted) ^a^ | *P* value (ANCOVA-adjusted) ^b^ |
| --- | --- | --- |
| *Roseburia* | ＜0.001 | 0.007 |
| *Roseburia_intestinalis* | ＜0.001 | 0.007 |
| *Roseburia_inulinivorans* | 0.400 | 0.775 |

ANCOVA, analysis of covariance. ^a)^P values were obtained from unadjusted between-group comparisons using the Mann-Whitney test; ^b)^P values were derived from an ANCOVA, adjusted for the use of the following concomitant antihyperglycemic therapy (as binary covariates): biguanides, DPP-4 inhibitors, insulin, alpha glucosidase inhibitors, sulfonylureas, thiazolidinediones, and glinides.

**Table S4 Summary of clinical parameters of the study participants before and after 12 weeks of treatment with stachyose**

|  | 0W | 12W | *P* value |
| --- | --- | --- | --- |
| Sex [male:female] | 16:3 | - | - |
| Age [years] | 50.00 (45.00,64.00) | - | - |
| Duration [years] | 7.00 (5.00, 11.00) | - | - |
| SBP [mmHg] | 122.00 (114.00, 135.00) | 120.00 (115.00, 128.00) | 0.250 |
| DBP [mmHg] | 78.00 (76.00, 89.00) | 80.00 (73.00, 83.00) | 0.142 |
| BMI [kg m^-2^] | 25.22 ± 2.41 | 24.90 ± 2.22 | 0.003 |
| HbA_1c_ [%] | 7.30 (6.50, 8.10) | 7.10 (6.40, 7.90) | 0.008 |
| UACR [mg g^-1^] | 169.30 (117.67, 236.05) | 97.77 (52.09, 168.95) | ＜0.001 |
| Urea [mmol L^-1^] | 6.56 ± 1.74 | 6.09 ± 1.59 | 0.129 |
| Creatinine [μmol L^-1^] | 67.17 ± 16.30 | 66.33 ± 16.78 | 0.603 |
| Uric acid [μmol L^-1^L] | 328.00 (294.00, 429.00) | 295.00 (272.00, 348.00) | 0.009 |
| eGFR [mL min^-1^ 1.73m^-2^] | 106.79 (93.80, 116.03) | 103.35 (94.50, 111.61) | 0.601 |
| TC [mmol L^-1^] | 3.75 ± 1.28 | 3.68 ± 1.11 | 0.798 |
| TG [mmol L^-1^] | 1.60 (1.08, 2.25) | 1.34 (0.94, 2.21) | 0.159 |
| HDL-C [mmol L^-1^] | 1.05 ± 0.30 | 1.11 ± 0.22 | 0.176 |
| LDL-C [mmol L^-1^] | 1.78 (1.14, 2.21) | 1.68 (1.22, 2.71) | 0.732 |

SBP, systolic blood pressure; DBP, diastolic blood pressure; BMI, body mass index; HbA1c, hemoglobin A1c; UACR, urine albumin-to-creatinine ratio; eGFR, estimated glomerular filtration rate; TC, total cholesterol; TG, triacylglycerol; HDL-C, high-density lipoprotein cholesterol; LDL-C, low-density lipoprotein cholesterol. Dates are expressed as mean ± s.d. or median (interquartile range, 25th-75th percentile). Comparisons were performed using a two-tailed paired Student’s *t*-test or the Wilcoxon signed-rank test, as appropriate.

**Table S5 Primer sequences of 16 rRNA (V3-V4)**

| Target Region | Primer | Sequence (5’-3’) |
| --- | --- | --- |
| 16 rRNA (V3-V4) | 338F | barcode + ACTCCTACGGGAGGCAGCA |
| 16 rRNA (V3-V4) | 806R | GGACTACHVGGGTWTCTAAT |

Barcode: the unique multiplexing identifier sequences used for each sample during library preparation.

**Table S6 Primer sequences**

| **Species** | **Primer** | **Sequence (5’→3’)** |  |
| --- | --- | --- | --- |
| **Bacteria** | *Roseburia intestinalis* | F: GCATGACCTGGTGTGAA | R: TTGGGCCGTGTCTCA |
|  | *Roseburia intestinalis β-fructosidase* | F: GACACTGTGGTTAGAGGAA | R: CCTGTATTGCTGTAAGTCTG |
|  | *Total bacteria* | F: ACTCCTACGGGAGGCAGCAG | R: ATTACCGCGGCTGCTGG |
| **Mouse** | *TNF-α* | F: GCTGAGCTCAAACCCTGGTA | R: CGGACTCCGCAAAGTCTAAG |
|  | *IL-6* | F: CCGGAGAGGAGACTTCACAG | R: TCCACGATTTCCCAGAGAAC |
|  | *β-actin* | F: GTGCTATGTTGCTCTAGATTCG | R: ATGCCACAGGATTCCATACC |
